# Adverse symptoms attributed to e-cigarettes over 6 months among participants of a randomized controlled trial testing nicotine free-base e-cigarettes for smoking cessation: secondary analysis of the ESTxENDS trial

**DOI:** 10.1093/ntr/ntag038

**Published:** 2026-02-20

**Authors:** Eva Güttinger, Angela Mosimann, Kali Tal, Anna Schoeni, Isabelle Jacot-Sadowski, Jean-Paul Humair, Susanne Pohle, Anja Frei, Seraina Netzer, Aurélie Berthet, Stéphanie Baggio, Reto Auer, Julian Jakob

**Affiliations:** Institute of Primary Health Care (BIHAM), University of Bern, Bern, Switzerland; Institute of Primary Health Care (BIHAM), University of Bern, Bern, Switzerland; Institute of Primary Health Care (BIHAM), University of Bern, Bern, Switzerland; Institute of Primary Health Care (BIHAM), University of Bern, Bern, Switzerland; Center for Primary Care and Public Health (Unisanté), University of Lausanne, Lausanne, Switzerland; Department of Primary Care Medicine, University Hospitals of Geneva, Geneva, Switzerland; Lung Center, Kantonsspital St. Gallen, St. Gallen, Switzerland; Epidemiology, Biostatistics and Prevention Institute, University of Zurich, Zurich, Switzerland; Institute of Primary Health Care (BIHAM), University of Bern, Bern, Switzerland; General Internal Medicine, Inselspital, University Hospital Bern, Bern, Switzerland; Center for Primary Care and Public Health (Unisanté), University of Lausanne, Lausanne, Switzerland; Institute of Primary Health Care (BIHAM), University of Bern, Bern, Switzerland; Institute of Psychology, University of Lausanne, Lausanne, Switzerland; Institute of Primary Health Care (BIHAM), University of Bern, Bern, Switzerland; Center for Primary Care and Public Health (Unisanté), University of Lausanne, Lausanne, Switzerland; Institute of Primary Health Care (BIHAM), University of Bern, Bern, Switzerland; Department of Paediatrics, Inselspital University Hospital Bern, Bern, Switzerland

**Keywords:** ENDS, e-cigarettes, symptoms, vaping, smoking, smoking cessation

## Abstract

**Introduction:**

People smoking tobacco cigarettes and switching to e-cigarettes report adverse symptoms attributed to e-cigarettes. We aimed at assessing the proportions and changes over 6 months in self-reported symptoms among participants of a large randomized controlled trial testing e-cigarettes for smoking cessation.

**Method:**

We included participants from the intervention group of the Efficacy, Safety and Toxicology of ENDS (ESTxENDS) randomized controlled trial. They received e-cigarettes, freebase nicotine e-liquids and smoking cessation counseling, phone follow-up at 1-, 2-, 4-, 8-week, and a visit at 6 months after target quit date. A set of predefined adverse symptoms experienced while vaping or smoking were systematically assessed at each contact. We used descriptive statistics and mixed models to report proportion of symptoms over time in exclusive e-cigarette users. We assessed the effect of symptoms on smoking re-initiation and the effect of duration of exclusive e-cigarette use on the resolution of symptoms in marginal structural models.

**Results:**

The intervention group included 622 participants, with a mean age of 40 (SD: 14) and 53% identified as men. After 1 week, the most commonly reported adverse symptoms among the 405 exclusive e-cigarette users were dry mouth (34%, 95 CI: 29%–39%), mouth/throat irritation (23%, 19%–27%), and cough (25%, 21%–29%). After 6 months, 256 exclusive e-cigarette users reported dry mouth (18%, 14%–23%), mouth/throat irritation (11%, 7%–15%), and cough (12%, 8%–16%). Marginal structural model revealed mouth/throat irritation led to smoking re-initiation, but continuing exclusive e-cigarette use resolved dry mouth in many.

**Conclusions:**

Adverse symptoms attributed to e-cigarettes are reported by fewer exclusive e-cigarette users over time. While continued e-cigarette use led to less dry mouth, mouth/throat irritation symptoms seemed to resolve because people experiencing symptoms switched back to smoking tobacco, while continuous exclusive e-cigarette users had less symptoms.

**Implications:**

E-cigarette use for smoking cessation can lead to adverse symptoms, such as dry mouth, mouth/throat irritation, and cough. The proportion of these symptoms, especially of dry mouth, was lower in participants still exclusively using e-cigarettes after 6 months than in people after one week of use. These findings are crucial for health care professionals who recommend e-cigarettes for smoking cessation, as they offer practical guidance on how to inform their patients about the possibility of these symptoms when shifting to e-cigarettes.

## Introduction

Electronic nicotine delivery systems (ENDS) or e-cigarettes are an effective,^1–6^ though controversial method of smoking cessation. Since e-cigarettes deliver 9- to 450-fold lower levels of toxic compounds than conventional tobacco cigarettes,^7^ and typically do not release toxic tobacco-specific nitrosamines or polycyclic aromatic hydrocarbons,^8^ many clinicians are now willing to counsel smokers to quit with e-cigarettes.^6^ However, e-cigarettes still release toxicants, contain propylene glycol (PG), vegetal glycerin (VG), aromas, and nicotine, which can all trigger symptoms when inhaled, including cough, dry mouth, and wheezing.^3,4,9^ Clincians and their patients need to know which symptoms are most common when considering e-cigarettes for smoking cessation and whether symptoms change over time. It is also relevant to know if adverse symptoms attributed to e-cigarettes (ASAEC) used for smoking cessation are as common and severe as symptoms triggered by smoking tobacco cigarettes.

Some evidence on prevalence and change of ASAEC can be found in smoking cessation clinical trials that tested the effects of e-cigarettes for smoking cessation; their findings suggest that respiratory, throat and mouth symptoms, and systemic symptoms including headache and dizziness are more frequent in the first days to weeks of vaping and decline over time (24–52 weeks) if users continue vaping.^3,4,10,11^ Observational studies reported varied results. Studies that included young e-cigarette users who never smoked tobacco, typically found that the risk of self-reported chronic cough, phlegm, bronchitis symptoms, wheeze, or self-reported diagnosis of asthma was higher in e-cigarette users.^9,12–14^ Another study that included older participants, who were never, former or current smokers found wheezing—a common respiratory symptom in asthma and chronic obstructive pulmonary disease (COPD)—was not associated with e-cigarette use, but with tobacco cigarette use.^15^ Another study found that participants with COPD experienced an improvement in respiratory symptoms when switching from tobacco cigarettes to e-cigarettes.^16^ Overall, there is a lack of systematic research on ASAEC over a prolonged period. Also, data on symptoms in ex-smokers or dual users are scarce and often not systematically assessed.

E-cigarettes vary widely in content of e-liquids and device to vaporize the e-liquid.^6^ Reports on symptoms need to clarify the content of the e-liquid and the device used by study participants to ensure adequate comparisons across datasets. For example, inhaled nicotine can cause respiratory irritation, which is often perceived as unpleasant.^17^ Nicotine can exist in protonated as well as in freebase form.^18^ Protonated nicotine (nicotine salt) is a form of nicotine that has been combined with an acid, making it less harsh and easier to inhale and therefore increases the appeal of vaping.^19^ In recent years, protonated nicotine has gained significant popularity. Also, the proportion of PG and VG in the e-liquid impacts symptoms, with a higher PG/VG ratio associated with more symptoms.^20,21^ The flavors in the e-liquid might also promote the occurrence of symptoms.^21–23^

We aimed at assessing the proportions and changes over 6 months in self-reported ASAEC among participants of a large randomized controlled trial (RCT) testing free-base nicotine e-cigarettes for smoking cessation. In this trial, most adhered to the e-liquid formulation and device combination provided in the trial for free, which enables to report on self-reported symptoms with this type of e-liquid and device when used for smoking cessation.

## Materials and methods

### Setting and design

We performed a secondary analysis of data from the intervention group of the Efficacy, Safety and Toxicology of Electronic Nicotine Delivery Systems as an aid for smoking cessation (ESTxENDS) trial. ESTxENDS is a multicenter RCT at five sites across Switzerland investigating the effectiveness and safety of e-cigarettes for smoking cessation. Participants were adult (≥18 years) smokers (≥5 cigarettes a day for at least 12 months) willing to quit smoking within the next three months. Details of the ESTxENDS trial have been published elsewhere.^5^ In brief, participants from the ESTxENDS trial were included from July 2018 to June 2021 and followed up for 6 months. Participants in the intervention group received standard-of-care (SOC) smoking cessation counseling, two e-cigarettes, and free access to e-liquids for 6 months. Participants in the control group received SOC smoking cessation counseling. Both groups were permitted to use pharmaceutical nicotine replacement therapy (NRT) at their own expense.

One to two weeks before the target quit date, participants attended a baseline visit, during which they were randomized into the control or intervention group. Study nurses called participants on their target quit date and 1, 2, 4, and 8 weeks thereafter. Participants attended an in-person visit at 6 months.

The local ethics committee at each participating site approved the trial and all participants granted written informed consent at baseline.^5^

### Study device and e-liquids

The study provided a third-generation e-cigarette (Innokin Endura T20-S starter kit) and “Alfaliquids” e-liquids produced by Gaïatrend in France. E-liquids components comprised PG, VG, freebase nicotine, alcohol, and flavors. The proportion of PG and VG was 76/24 for all e-liquids. Participants could choose free-base nicotine concentrations of 0, 6, 11, or 19.6 mg/mL and in tobacco, menthol, or fruity flavors. Study nurses instructed participants on how the e-cigarette is set up, how to use and charge the device, and how to change e-liquid and coil. Participants could try and mix different flavors and nicotine-strengths based on their preferences, previous cigarette use and adverse symptoms attributed to nicotine. Study nurses advised 19.6 mg/mL e-liquids in case of high or very high nicotine dependence and 11 mg/mL for medium and low dependence. Study nurses instructed participants on vaping techniques. To lower the harshness, they advised to inhale slowly and hold the vapor in the mouth for about 3 s before inhaling it into the lungs (mouth-to-lung) instead of inhaling directly into the lungs (direct-to-lung). Participants were permitted to purchase and use e-liquids other than those provided by the study.^5^

### Participants and exposure

At each contact, we actively queried participants about their e-cigarette use and tobacco cigarette smoking in the 7 days prior to the contact (self-reported). We divided participants into four categories by exposure: “tobacco and e-cigarette abstainers” (no smoking or vaping in the past 7 days); “exclusive smokers” (smoking and no vaping in the past 7 days); “exclusive e-cigarette users” (vaping but no smoking in past 7 days); and “dual users” (smoking and vaping in the past 7 days). We focused on ASAEC, so we restricted the analyses to exclusive e-cigarette users from the intervention group in this secondary analyses of the ESTxENDS trial. We stratified analyses to participants that were exclusive e-cigarette users. For secondary analyses, we studied outcomes in continuous exclusive e-cigarette users (participants reporting exclusive e-cigarette use over all available study visits) and dual users.

### Outcomes: adverse symptoms attributed to e-cigarettes

Primary outcomes were symptoms at 1, 2, 4, and 8 weeks and after 6 months among participants from the ESTxENDS intervention group who were e-cigarette users. We assessed ASAEC through a structured questionnaire administered systematically during all follow-up phone calls and clinical visits. The following predefined set of symptoms were systematically assessed at each contact: dry mouth, mouth/throat irritation, cough, shortness of breath, headache, dizziness, heart palpitations/tachycardia, and/or “Other” symptoms (via open text question, participants could mention any symptom not assessed in the previous questions). We based our choice of symptoms on our review of three studies on the topic,^3,11,24^ choosing the six most frequently reported ASAEC. We told participants they should report these symptoms only if they experienced them during or immediately after vaping: “Since you last visited or spoke with us on the phone, have you had one or more of the following symptoms that you associated with the vaping device (e-cigarette)?” If participants answered yes, we asked if they had experienced the symptom within the past 24 h. If yes, we asked them to describe its intensity on a scale of 1 (very weak) to 6 (very strong) and rate how strongly they felt this symptom gave cause for ceasing e-cigarette or tobacco cigarette use on a 0 (not at all) to 5 (absolutely) on a Likert scale. We asked the same questions on symptoms occurring during the smoking of a cigarette.

### Covariables

Covariables included gender (woman, man, other), age, educational grade, current working status, marital status, smoking history including packyears, cannabis use within the last 30 days, alcohol consumption (AUDIT-C Score),^25^ self-reported respiratory disease (COPD, chronic bronchitis, and asthma) e-liquid components such as nicotine concentration and flavors and puffs/day.

### Statistical methods/data analysis

We used descriptive statistics to present baseline characteristics of our sample and prevalence and intensity of ASAEC at each visit, by current vaping and smoking status. We present the occurrence of symptoms between week 1 (the first week participants used e-cigarette) and the following visits. Sensitivity analyses included symptoms among continuous exclusive e-cigarette users over 6 months (participants reporting exclusive e-cigarette use over all available study visits). We also present symptoms attributed to cigarettes in all smokers at baseline to allow the reader to compare the magnitude of symptoms attributed to e-cigarettes with previously experienced symptoms while smoking.

We then applied multivariable adjusted mixed models to compare proportions of ASAEC in exclusive e-cigarette users at week 1 (the first week participants used e-cigarette) and all following visits. We correlated random participant-specific intercepts and slopes to account for within-individual correlation of repeated measures and to model individual departures from the trajectories determined by the fixed effects. Age and visit were modeled as fixed effects and included in our multivariable adjusted model as random effects to identify time-dependent trends. We used inverse probability of attrition weighting including study site, baseline demographic variables (age, sex, relationship status, employment situation, educational status), smoking history (cigarettes per day and number of previous quitting attempts), alcohol and cannabis use, self-reported respiratory disease, atopic disease, arterial hypertension, depression score, nicotine concentration, and flavorings of e-liquids and puff/days as predictors.

As vaping and smoking are time-dependent, we performed exploratory, secondary post-hoc analyses in a causal framework. We tested (a) if ASAECs led people reporting exclusive e-cigarette use to tobacco re-initiation (exclusive e-cigarette use to exclusive smoking or dual use), censoring participants at the first change in self-reported vaping status from vaping to not vaping, and (b) if days of vaping (cumulative estimate based on self-reported vaping status at each phone call, adding up the days of vaping between phone calls) led to resolution of symptoms by fitting marginal structural models to deal with time-dependent confounder-mediators with the same covariables as presented above, using a counterfactual statistical approach (see Appendix Methods).

Finally, we assessed symptoms attributed to cigarettes in exclusive smokers from the control group cross-sectionally over 6 months to see whether symptoms change over time and to compare the results with ASAEC over time.

Tests of statistical significance were two-tailed; alpha level was 0.05. All analyses were conducted with Stata version 18 (StataCorp LP, College Station, TX, USA).

## Results

### Participants

We included all 622 participants from the intervention group; mean age at study entry was 40 (14) years; 53% identified as men. Median number of cigarettes smoked daily was 15; median number of packyears was 14. Self-reported past medical conditions included asthma (12%), COPD (4%), and chronic bronchitis (4%) (see Table 1).

**Table 1:** Baseline characteristics of the 622 participants of the intervention group (subgroup: week 1 exclusive e-cigarette users).

Characteristics	Participants	W1 exclusive e-cigarette users
Participants, *n*	622	405
Age, Mean (SD); [range]	40 (14); [18–79]	40 (13); [19–79]
Gender		
Women no. (%)	289 (47)	184 (45)
Men no. (%)	331 (53)	221 (55)
Other no. (%)	0 (0)	0 (0)
Education		
Primary/other/none no. (%)	50 (8)	32 (8)
Secondary no. (%)	398 (64)	250 (62)
Tertiary no. (%)	172 (28)	123 (30)
Work situation		
Work/house no. (%)	446 (72)	305 (75)
In formation no. (%)	57 (9)	33 (8)
Looking for a job/other no. (%)	117 (19)	67 (17)
Marital status		
Single no. (%)	346 (56)	217 (54)
Married/registered partnership no. (%)	169 (27)	123 (30)
Widow/divorced/dissolved no. (%)	105 (17)	65 (16)
Smoking history		
No. of cigarettes smoked daily Median (p25, p75); [range]	15 (10, 20); [5–60]	15 (10, 20); [5–45]
Age when started smoking Median (p25, p75); [range]	16 (15, 18); [8–46]	16 (15, 18); [8–32]
No. of previous quit attempts Median (p25, p75); [range]	2 (1, 3); [0–50]	2 (1, 3); [0–50]
Fagerström score Median (p25, p75); [range]; Mean (SD)	4 (3, 6); [0–10]; 4.3 (2.3)	4 (3, 6), [0–10]; 4.2 (2.3)
Expired carbon monoxide level Median (p25, p75); [range]	20 (13, 29); [0–128]	19 (13, 29); [0–128]
Packyears^a^ Median (p25, p75); [range]	14 (7, 28); [0–132]	14 (7, 25); [0–106]
Nicotine concentration (mg/mL); Mean (SD)		13 (5)
Flavors		
Fruity, *N* (%)		101 (25%)
Tobacco, *N* (%)		97 (24%)
Menthol, *N* (%)		35 (9%)
Other flavors, *N* (%)		1 (<1%)
Flavor mix, *N* (%)		169 (42%)
Puffs a day		
1–5, *N* (%)		7 (2%)
6–50, *N* (%)		143 (37%)
>50, *N* (%)		238 (61%)
Substance use		
Alcohol: AUDIT C-Score Median (p25, p75); [range]	4 (2–5); [0–12]	4 (2–5); [0–11]
Cannabis used in last 30 days no. (%)	109 (18)	68 (17)
Health status		
COPD no. (%)	25 (4)	13 (3)
Chronic bronchitis no. (%)	22 (4)	11 (3)
Asthma no. (%)	77 (12)	51 (13)
CAT score total Median (p25, p75); [range]	8 (5–12); (0–28)	8 (4–12); (0–28)
CAT score question 1—cough Median (p25, p75); [range]	2 (1–3); (0–5)	2 (1–3); (0–5)
CAT score question 2—phlegm Median (p25, p75); [range]	1 (0–2); (0–5)	1 (0–2); (0–5)

^a^Number of packs of cigarettes smoked per day multiplied by the number of years the participant has smoked.

### Exclusive e-cigarette users cross-sectionally

#### Adverse symptoms attributed to e-cigarettes

At baseline, when they were still exclusive smokers, most participants reported symptoms attributed to cigarettes: dry mouth, mouth/throat irritation, cough, and dizziness. Less frequent symptoms included shortness of breath, headache, palpitations/tachycardia, and “Other” symptoms described in open text (see Table 2 for all numbers, and Appendix Figure 1).

**Table 2:** Symptoms attributed to e-cigarettes in exclusive e-cigarette users cross-sectionally at every visit.

	Symptoms attributed to cigarettes	Symptoms attributed to e-cigarettes				
	BL *n* = 622	W1	W2	W4	W8	M6
Total exclusive e-cigarette users, *N*		405	396	386	351	256
Dry mouth, *N* (%) Intensity (p25, p75); [range: 1–6] Reason to stop (p25, p75); [range: 0–5]	310 (50%) 3.0 (2.0; 4.0) 1.0 (0.0; 4.0)	139 (34%) 3.0 (2.0; 4.0) 0.0 (0.0; 1.0)	130 (33%) 3.0 (2.0; 4.0) 0.0 (0.0; 0.0)	110 (28%) 3.0 (2.0; 4.0) 0.0 (0.0; 0.0)	74 (21%) 3.0 (2.0; 4.0) 0.0 (0.0; 1.8)	47 (18%) 2.0 (1.0; 3.0) 0.0 (0.0; 0.0)
Mouth/throat irritation, *N* (%) Intensity (p25, p75); [range: 1–6] Reason to stop (p25, p75); [range: 0–5]	193 (31%) 3.0 (2.0; 4.0) 4.0 (2.0; 5.0)	93 (23%) 3.0 (2.0; 4.0) 0.0 (0.0; 1.0)	95 (24%) 30. (2.0; 3.5) 0.0 (0.0; 1.0)	71 (18%) 3.0 (2.0; 4.0) 0.0 (0.0; 2.0)	57 (16%) 3.0 (2.0; 3.0) 0.0 (0.0; 1.8)	29 (11%) 3.0 (2.0; 3.0) 0.0 (0.0; 0.0)
Cough, *N* (%) Intensity (p25, p75); [range: 1–6] Reason to stop (p25, p75); [range: 0–5]	168 (27%) 3.0 (2.0; 4,0) 5.0 (3.0; 5.0)	103 (25%) 3.0 (2.0; 3.0) 0.0 (0.0; 1.0)	74 (19%) 2.0 (1.0; 3.0) 0.0 (0.0; 0.0)	52 (13%) 3.0 (2.0; 3.0) 0.0 (0.0; 0.0)	48 (14%) 2.0 (2.0; 3.0) 0.0 (0.0; 2.0)	31 (12%) 2.0 (1.0; 2.0) 0.0 (0.0; 0.5)
Shortness of breath, *N* (%) Intensity (p25, p75); [range: 1–6] Reason to stop (p25, p75); [range: 0–5]	94 (15%) 3.0 (2.0; 5.0) 5.0 (4.0; 5.0)	13 (3%) 4.0 (2.5; 5.0) 0.0 (0.0; 3.5)	10 (3%) 2.0 (2.0; 2.8) 0.0 (0.0; 0.0)	10 (3%) 3.0 (2.5; 4.0) 0.0 (0.0; 3.8)	6 (2%) 3.0 (2.2; 3.0) 0.0 (0.0; 0.8)	4 (2%) 4.0 (4.0; 4.0) 0.0 (0.0; 0.0)
Headache, *N* (%) Intensity (p25, p75); [range: 1–6] Reason to stop (p25, p75); [range: 0–5]	85 (14%) 3.0 (2.0; 4.0) 5.0 (3.0; 5.0)	27 (7%) 3.0 (2.0; 3.0) 0.0 (0.0; 4.0)	23 (6%) 4.0 (2.8; 4.0) 0.5 (0.0; 3.0)	20 (5%) 2.5 (1.0; 4.0) 0.0 (0.0; 0.8)	17 (5%) 2.0 (1.2; 2.8) 0.0 (0.0; 0.0)	11 (4%) 2.5 (2.0; 3.0) 0.0 (0.0; 0.0)
Dizziness, *N* (%) Intensity (p25, p75); [range: 1–6] Reason to stop (p25, p75); [range: 0–5]	168 (27%) 3.0 (2.0; 4.0) 2.0 (0.0; 5.0)	16 (4%) 3.0 (3.0; 3.2) 0.0 (0.0; 4.2)	12 (3%) 3.0 (3.0; 4.0) 0.0 (0.0; 2.0)	10 (3%) 3.0 (1.0; 4.0) 0.0 (0.0; 2.0)	6 (2%) 4.0 (3.0; 5.0) 2.5 (0.0; 5.0)	3 (1%) n/a n/a
Palpitations/tachycardia, *N* (%) Intensity (p25, p75); [range: 1–6] Reason to stop (p25, p75); [range: 0–5]	61 (10%) 3.5 (2.0; 5.0) 5.0 (2.0; 5.0)	8 (2%) 3.0 (1.5; 5.5) 0.0 (0.0; 0.5)	11 (3%) 2.0 (1.8; 3.2) 0.0 (0.0; 2.0)	12 (3%) 3.0 (3.0; 3.0) 0.0 (0.0; 1.0)	7 (2%) 2.0 (1.0; 3.0) 0.0 (0.0; 0.0)	6 (2%) n/a n/a
Other, *N* (%) Intensity (p25, p75); [range: 1–6] Reason to stop (p25, p75); [range: 0–5]	50 (8%) 4.0 (3.0; 5.0) 4.5 (3.0; 5.0)	26 (6%) 3.0 (2.8; 4.0) 0.0 (0.0; 2.0)	19 (5%) 3.0 (2.0; 5.0) 0.0 (0.0; 4.0)	17 (4%) 4.0 (2.0; 5.0) 0.0 (0.0; 3.0)	15 (4%) 3.0 (3.0; 4.0) 2.0 (0.0; 3.0)	20 (8%) 2.0 (2.0; 5.0) 0.0 (0.0; 0.0)

BL = Baseline, *N* = number, W = week, M = month.

At week 1, 553 participants completed the questionnaire on ASAEC: 405 (73%) exclusively used e-cigarettes; 126 (23%) were dual users; 4 (1%) smoked exclusively; and 18 (3%) abstained from both tobacco and e-cigarette (see Appendix Figure 2). Exclusive e-cigarette users most often reported dry mouth, mouth/throat irritation, and cough. They less frequently reported headache, dizziness, shortness of breath, heart palpitations/tachycardia, and “Other” via open text (see Table 2 for all numbers and results from W2 to W8, and Appendix Figure 3). They infrequently reported “Other” symptoms; these were heterogeneous, and the most common were oral problems (burning tongue, mucosal irritation, changes in taste perception), abdominal symptoms (nausea, stool irregularities, flatulence, epigastric pain), and thoracic symptoms (chest pain/pressure, wheezing; see Appendix).

At month 6, 519 participants completed the questionnaire; 256 (49%) participants reported exclusive e-cigarette use; 96 (18%) were dual users; 105 (20%) were smokers; and 62 (12%) were abstainers (see Appendix Figure 2). Among exclusive e-cigarette users, these were the most common ASAEC: dry mouth; mouth/throat irritation; and cough (see Table 2 for all numbers and results from W2 to W8, and Appendix Figure 3).

#### Intensity and reason to stop

In the cross-section of exclusive e-cigarette users, median intensity of symptoms at week 1 was 3 (range: 1–6) except for shortness of breath, which was 4. At month 6, the intensity of dry mouth and cough was 2 and mouth/throat irritation was 3 (see Table 2).

Exclusive e-cigarette users did not rate their symptoms as a reason to stop vaping (see Table 2).

#### Contrast between ASAEC at week 1 and month 6 in exclusive e-cigarette users

Our adjusted mixed model showed that the occurrence of dry mouth, mouth/throat irritation, and cough were reported less frequently after 6 months compared to week 1 (see Table 3).

**Table 3:** Mixed model comparing symptoms at weeks 2, 4, 8, and month 6 to symptoms at week 1 in exclusive e-cigarette users.

	W2	W4	W8	M6
Dry mouth coefficient % (95% CI) *p*-value	−0.038 (−0.093 to 0.174) .179	−0.062 (−1.22 to −0.002) .042	−0.138 (−0.202 to −0.075) <.001	−0.148 (−0.231 to −0.066) <.001
Mouth/throat irritation coefficient % (95% CI) *p*-value	0.022 (−0.039 to 0.085) .473	−0.025 (−0.085 to 0.034) .402	−0.043 (−0.106 to 0.02) .177	−0.088 (−0.159 to −0.016) .016
Cough coefficient % (95% CI) *p*-value	−0.057 (−0.114 to −0.0002) .049	−0.107 (−0.163 to −0.050) <.001	−0.108 (−0.171 to −0.044) .001	−0.157 (−0.233 to −0.082) <.001
Headache % (95% CI) *p*-value	−0.018 (−0.049 to 0.012) .228	−0.007 (−0.044 to 0.030) .712	−0.003 (−0.043 to 0.036) .880	0.015 (−0.060 to 0.030) .502

Interpretation: 1 means experiencing the symptom, 0 means not experiencing it. A coefficient of −0.1 can be interpreted as 10% less of the observed population at the corresponding visit experienced the symptom. Adjusted for age, gender, education, work situation, cigarettes/day, cannabis used in last 30 days, AUDIT C-Score, Health status (COPD, Asthma), Nicotine concentration in e-liquids, flavors in e-liquids, puffs/day.

#### Exploratory analyses of the effects of ASAEC on tobacco re-initiation

The first marginal structural model showed that experiencing mouth/throat irritation during a visit increased the participant’s odds of switching from exclusive e-cigarette use to smoking tobacco or dual (OR 1.7, 95% CI: 1.2 to 2.6). Dry mouth or coughing did not lead to tobacco re-initiation (Table 4).

**Table 4:** Effect of symptoms on tobacco re-initiation, results from a marginal structural model.

Effect of the following symptoms (predictors) on tobacco re-initiation	Odds ratio (95 CI) for vaping cessation and tobacco re-initiation	*p*-value
Dry mouth	0.9 (0.6–1.3)	.56
Mouth/throat irritation	1.7 (1.2–2.6)	.008
Cough	1.3 (0.9–2.1)	.2

Interpretation: the OR for vaping cessation and tobacco re-initiation (switching from exclusive e-cigarette use to smoking or dual use) was 1.7 in exclusive e-cigarette users that experienced mouth/throat irritation. Other symptoms not included as too few incidences and changes.

#### Exploratory analyses of the effects of ASAEC on changes in vaping habits

The second marginal structural model showed that increased vaping duration led to resolution of dry mouth (OR 1.008, 95% CI: 1.004 to 1.014 per day of vaping). Mouth/throat irritation or coughing did not resolve with increasing vaping duration (Table 5 and Appendix Figure 4).

**Table 5:** Effect of duration of vaping on symptoms resolution, results from a marginal structural model.

Symptom resolution (outcome)	Odds ratio (95 CI) of symptoms resolution	*p* value
Dry mouth resolution	1.009 (1.004–1.014)	<0.001
Mouth/throat irritation resolution	1.003 (0.997–1.009)	0.3
Cough resolution	1.003 (0.996–1.009)	0.4

Interpretation: the OR for resolution of dry mouth (experiencing dry mouth at a visit, and resolution at the next visit) was 1.009 per day of vaping in exclusive e-cigarette users. Also see Appendix Figure 8. Other symptoms not included as too few incidences and changes.

### ASAEC in continuous exclusive e-cigarette users

After 6 months, 181 (35%) participants were continuous exclusive e-cigarette users and most often reported symptoms were dry mouth, mouth/throat irritation, and cough (see Appendix Table 1 and Appendix Figure 5).

### Adverse symptoms attributed to cigarettes in exclusive smokers from the control group

At baseline, 619 participants in the control group answered the questionnaire on symptoms attributed to cigarettes. Following symptoms were most commonly reported: dry mouth (40%), mouth/throat irritation (29%), cough (26%), and dizziness (25%). After one week there were 198 exclusive smokers with the following symptoms: dry mouth (20%), mouth/throat irritation (11%), cough (11%), and dizziness (16%). At month 6, 237 participants were exclusive smokers. 27% reported dry mouth, 16% reported mouth/throat irritation, 14% reported cough, and 14% reported dizziness (see Appendix Table 5 and Appendix Figure 6).

## Discussion

### Key results/interpretation

Our study of self-reported ASAEC over 6 months among participants of a RCT that provided free-base e-cigarettes for smoking cessation revealed that dry mouth, mouth/throat irritation, and cough were the most often reported symptoms; all of these declined over time. While dry mouth did not lead to subsequent tobacco re-initiation, increased exclusive vaping duration led to resolution of dry mouth, indicating this ASAEC is likely to decrease over time. The opposite was true for mouth/throat irritation. The presence of this ASAEC led to tobacco re-initiation and did not resolve with increased exclusive vaping duration, indicating descriptive results and mixed models approaches might be biased. Participants not experiencing ASAEC might thus stick to vaping, while participants with uncomfortable adverse symptoms switch back to tobacco smoking, leading to the misinterpretation that the prevalence of adverse symptoms decreases over time in cross-sectional analyses of currently exclusive e-cigarette smokers.

Few trials have described in detail the occurrence of ASAEC over time.^3,4,10,11^ A RCT that compared free-base e-cigarettes to NRT found throat/mouth irritation was more frequent in the e-cigarette group. They also found a greater decline in cough and phlegm production in the e-cigarette group after 52 weeks, possibly because the e-cigarette group had a higher smoking cessation rate.^4^ Another RCT that compared free-base e-cigarettes with nicotine to e-cigarettes without nicotine found that cough, dry mouth, shortness of breath, throat irritation, and headache decreased significantly over time in all groups.^3^

Decline in ASAEC may be influenced by many factors, including the strategies e-cigarette users develop to reduce symptoms. E-cigarette users seem to “learn” how to reduce their symptoms. For example, vaping counselors advise beginners to inhale “mouth-to-lung,” holding the vapor in the mouth before inhaling to reduce the intensity of the experience because “direct-lung inhalation” can lead to cough.^26^ Study participants in ESTxENDS were instructed to vape “mouth-to-lung,” so our findings apply to people using this technique only. E-cigarette users can also reduce symptoms by changing nicotine concentrations, flavors, or the proportion of PG/VG. They could use protonated nicotine (“nicotine salts”), which causes less airway irritation than the free-base nicotine we used in this study. Another explanation for the observed decline in ASAEC over time might be a general time effect. Nevertheless, we found no such decline in symptoms attributed to cigarettes in exclusive smokers from the control group.

Since smoking cigarettes irritates the airways despite many additives,^27^ the symptoms of those who switch to vaping may decline over time. We found that exclusive e-cigarette users had fewer and less severe symptoms than the symptoms attributed to cigarettes they had reported at baseline.

### Limitations

This study has four main limitations. First, systematically assessing symptoms might encourage participants to over-report their symptoms. Symptoms are subjective outcomes and therefore prone to bias. Participants who are informed about potential symptoms are more likely to report experiencing them. Mitigating possibilities such as blinding was not possible. However, we found a decreasing dynamic of ASAEC over time which is representative even if symptoms are over-estimated. Second, while we predefined a set of ASAEC based on previous findings, the set of questions has not undergone rigorous evaluation. When we launched the trial in 2018, no formal questionnaire for ASAEC existed. In the meantime, the Respiratory Symptoms Experience Scale,^28^ a questionnaire querying for similar symptoms with similar methods, the MECEQ Questionnaire (focus on positive or negative vaping effects, not specific symptoms)^29^ and the respiratory symptom index from the PATH study (focus on cough/wheezing)^30,31^ have been developed and validated. Ongoing studies are using this questionnaire to systematically assess ASAEC and we hope the similarity of the question will help future comparisons of symptoms reporting across studies.^32^ Third, because our response rate was 89% at week 1 and 83% at month 6, it is possible that participants who didn’t respond to the questionnaire had more ASAEC. Still, this follow-up rate is one of the highest among the studies published on symptoms. Fourth, vaping/smoking status was not verified using biochemical measures. Participants were asked to report their smoking/vaping habit over the past 7 days which we assumed they would remember correctly, even though some misreporting cannot be excluded. Participants were not assigned to a predefined group nor were they asked to adhere to the group. At each visit, participants were categorized to a group based on their current smoking/vaping status. We assessed ASAEC only if participants reported currently using e-cigarettes. Finally, our results are limited to e-liquids containing free-base nicotine, a 70/30 PG/VG ratio and used with one device provided in this trial; the proportions reporting symptoms might be lower with protonated nicotine e-cigarettes or with other PG/VG combinations and devices. Also, results apply to the population included in the ESTxENDS trial conducted in Switzerland.

## Conclusions

In this study on participants of a RCT using free-base e-cigarettes as a smoking cessation tool, we found that e-cigarette users initially reported dry mouth, mouth/throat irritation, and cough in a proportion similar to what was reported symptoms attributed to cigarettes at baseline. After 6 months, these ASAEC declined significantly in exclusive e-cigarette users, especially dry mouth. Other symptoms were less often reported over time, but this finding is likely to be biased by people not experiencing symptoms that persist as exclusive e-cigarette users, while participants experiencing symptoms switch back to tobacco, and are thus not considered in subsequent analyses in exclusive e-cigarette users.

Understanding that initial discomfort is common and, for some adverse symptoms, is likely to diminish over time is crucial for clinicians to manage their patients’ expectations during smoking cessation counseling. Unexplained discomfort during a quitting attempt can erode motivation and lead to a return to cigarette smoking, as observed in mouth/throat irritation. Clinicians can inform their patients that ASAEC such as dry mouth are usually transient and help the patients to anticipate and tolerate the symptoms which leads to higher adherence and minimizes the risk of a relapse. However, clinicians may inform that “mouth/throat irritation” might not abate, clarifying that these were the findings in a study which offered free-base nicotine e-liquids and not nicotine salts, where these symptoms might occur less often. Aligned with patient’s preferences toward ongoing nicotine use, some might include a plan for possible cessation of e-cigarette as the potential goal is cessation of e-cigarettes in a second step.^33,34^

The findings of this study on the natural evolution of ASAEC can thus help health professionals to effectively counsel and accompany smokers in their quitting attempt with regards to symptoms.

## Supplementary Material

Appendix_ENDS_symptoms_14_02_26_ntag038

## Data Availability

Data are available from the corresponding author upon request.
