# Supplementary material for: Adverse symptoms attributed to e-cigarettes over 6 months among participants of a randomized controlled trial testing nicotine free-base e-cigarettes for smoking cessation: secondary analysis of the ESTxENDS trial
Source: Nicotine Tob Res. 2026 Feb 20;28(8):1284–92. doi: 10.1093/ntr/ntag038 (PMC13389526; doi:10.1093/ntr/ntag038)
Supplement: Appendix_ENDS_symptoms_14_02_26_ntag038 [file appendix_ends_symptoms_14_02_26_ntag038.docx]

# Adverse symptoms attributeD to e-cigarettes over six months among participants of a randomized controlled trial testing nicotine freebase e-cigarettes for smoking cessation – Secondary analysis of the ESTxENDS trial

Table of contents

[Adverse symptoms attributeD to e-cigarettes over six months among participants of a randomized controlled trial testing nicotine freebase e-cigarettes for smoking cessation – Secondary analysis of the ESTxENDS trial 1](#_Toc221932270)

[Methods 3](#_Toc221932271)

[ENDS and E-Liquids 3](#_Toc221932272)

[Participants 4](#_Toc221932273)

[Marginal structural models to deal with time-dependent confounder-mediators (TDCMs). 4](#_Toc221932274)

[Results: 5](#_Toc221932275)

[Dual users 5](#_Toc221932276)

[Continuous dual users 6](#_Toc221932277)

[Contrast between symptoms at Week 1 and Month 6 in continuous dual users 6](#_Toc221932278)

[Other symptoms 6](#_Toc221932279)

[Discussion 9](#_Toc221932280)

[Dual users 9](#_Toc221932281)

[Appendix Figures 10](#_Toc221932282)

[Appendix Figure 1) Symptoms attributed to smoking at Baseline – Mean (Percentage), N=622 10](#_Toc221932283)

[Appendix Figure 2) Number of participants and their smoking / vaping status over time 11](#_Toc221932284)

[Appendix Figure 3) Symptoms attributed to e-cigarettes in current exclusive e-cigarette users - Percentage 12](#_Toc221932285)

[Appendix Figure 4) Effect of days of vaping on resolution of dry mouth (results from a marginal structural model) 13](#_Toc221932286)

[Appendix Figure 5) Symptoms attributed to e-cigarettes in continuous exclusive e-cigarette users – Percentage 14](#_Toc221932287)

[Appendix Figure 6) Symptoms attributed to cigarettes in exclusive smokers cross-sectionally (control group) – Percentage 15](#_Toc221932288)

[Appendix Figure 7) Symptoms attributed to e-cigarettes in current dual users – Percentage 16](#_Toc221932289)

[Appendix Figure 8) Symptoms attributed to e-cigarettes in continuous dual users – Percentage 17](#_Toc221932290)

[Appendix Table 1) Symptoms attributed to e-cigarettes in continuous exclusive e-cigarette users 18](#_Toc221932291)

[Appendix Table 2) Baseline characteristics of the 622 Participants of the intervention group (subgroup: Week 1 dual users) 19](#_Toc221932292)

[Appendix Table 3) Symptoms attributed to e-cigarettes in dual users cross-sectionally at every visit 22](#_Toc221932293)

[Appendix Table 4) Symptoms attributed to e-cigarettes in continuous dual users 23](#_Toc221932294)

[Appendix Table 5) Symptoms attributed to cigarettes in exclusive smokers cross-sectionally (control group) 24](#_Toc221932295)

[Appendix Table 6) Choice of e-liquids (flavors/ nicotine concentrations) and puffs/day in exclusive e-cigarette users 25](#_Toc221932296)

[Appendix Table 7) Main Symptoms by flavoring use 27](#_Toc221932297)

[Appendix Table 8) Comparison between participants lost to follow up versus not lost to follow up 28](#_Toc221932298)

[References 30](#_Toc221932299)

# Methods

## ENDS and E-Liquids

We used the Alphaliquid e-liquids produced by Gaïatrend in France (https://www.gaiatrend.fr/en/), which comply with the strict quality criteria set by Agence Française de Normalisation (AFNOR). Available nicotine concentrations were 0 mg/ml, 6 mg/ml, 11 mg/ml, and 19.6 mg/ml. Available flavorings were FR-4 (tobacco flavor) and FR-M (tobacco flavor), FRESH MINT (menthol flavor), RASPBERRY#2 (fruity flavor), RED FRUITS (fruity flavor) or GREEN APPLE (fruity flavor). For all e-liquids, the proportion of propylene glycol to vegetable glycerin was 76/24. E-liquids contained propylene glycol, vegetable glycerin, medical-quality free-base nicotine, alcohol, and flavorings. To avoid financial conflicts of interest with the manufacturer, the study team paid the factory price to Gaïatrend for the e-liquids and paid for the shipping of the e-liquids to the study centers, which were then given for free to study participants.

The Endura T20-S kit, produced in China by “Innokin”, came in a user packet with a 1,500 mAh internal Li-Po battery, a Prism S Coil (0.8 ohm, 16-18W) atomizer, a spare drip tip, a micro-USB DC 5V/1A cable and an instruction manual in French or German. Devices came in 5 colors; participants could choose between black, purple, grey, red, and blue. To avoid financial conflicts of interest with the manufacturer, the study team paid the factory price to Innokin for the kits and spare coils and paid for the shipping of the e-liquids to the study centers, which were then given for free to study participants. [1] (supplementary Appendix)

## Participants

For secondary analyses, we studied outcomes in continuous exclusive e-cigarette users (participants reporting exclusive e-cigarette use over all available study visits), in dual users (smoking and vaping in the past seven days), and continuous dual users (participants reporting dual use over all available study visits).

**Example 1**: A participant was an exclusive e-cigarette user at Week 1 and was also an exclusive e-cigarette user at Week 2, then this person counts as continuous exclusive e-cigarette user at the Week 2 visit. The same would be for a person that was not available for visit at Week 1, but states at Week 2 that he/she exclusively used e-cigarettes since the last visit (which would then be the baseline visit).

**Example 2**: A participant was an exclusive e-cigarette user at Week 1 and 2, but no data was available for Week 4. At the 6 Month visit, the participant claims to have used only e-cigarettes since the last visit (in his/her case the last visit was at Week 2), then this participant also counts as continuous e-cigarette user at the Month 6 visit.

The same applies analogously for dual users. [2] (supplementary Appendix)

## Marginal structural models to deal with time-dependent confounder-mediators (TDCMs).

In observational studies with time-varying exposures, standard adjustment to control confounding may be biased if important time-dependent confounders of exposure also mediate its effects.[3] Marginal structural models (MSMs) use inverse-probability-of-treatment weights (IPTWs) to obtain exchangeable pseudo-populations of exposed and unexposed that can be compared without adjustment. To reduce bias from potentially informative attrition, MSMs also commonly use inverse probability of censoring weights (IPCWs). As recommended, we used both weights used in “stabilized” form to reduce variability. The final weights, obtained as the product of the stabilized IPTWs and IPCWs, were then used in an unadjusted population averaged repeated measures model to estimate the causal effect of adverse symptoms on tobacco re-initiation and, separately, for days of vaping on resolution of symptoms.[4] The rationale for this procedure is that by using the combined stabilized weights to make predictors exchangeable, we avoid having to adjust away the indirect effects of experiencing a symptom or not / days of vaping mediated by the proposed TDCMs. A common drawback of MSMs is loss of precision, as compared to covariable adjustment.

To estimate both the numerator and denominator of the stabilized weights, we included the same set of covariables as in the main, mixed models. Models used to estimate the weight denominators also included lagged values of the proposed TDCMs. The final weighted MSMs used robust standard errors.

# Results:

## Dual users

At Week 1, 126 participants were dual users (see Appendix Table 2 for baseline characteristics). Among them, the following vaping-associated symptoms were most reported: dry mouth, mouth/throat irritation, cough, and headache (see Appendix Table 3 for all numbers and results from W2-W8, and Appendix Figure 7). The 96 dual users at Month 6 most often reported dry mouth, mouth/throat irritation, and cough (see Appendix Table 3 for all numbers and results from W2-W8).

## Continuous dual users

Only 11 participants (2%) were continuous dual users after 6 months. Participants reported dry mouth, mouth/throat irritation, and cough (see Appendix Table 4, Appendix Figure 8).

## Contrast between symptoms at Week 1 and Month 6 in continuous dual users

As only 11 participants reported dual use over all study visits by Month 6, we refrained from performing multivariable adjusted models in this population.

## Other symptoms

W1

| Thoracic | Pressure on chest, lung irritation | 5 |
| --- | --- | --- |
| Abdominal | Constipation, stool irregularities | 2 |
| Nausea |  | 4 |
| Enoral | Burning tongue, irritation, sharpness on tongue, aphthae, throat irritation, salivation, swallowing | 7 |
| Fatigue |  | 1 |
| Psychological | Increased irritability, emptiness | 2 |
| Sneezing |  | 1 |
| Hiccups |  | 1 |
| Sweating |  | 1 |
| Disgust |  | 1 |

W2

| Thoracic | Pressure on chest, burning lungs | 2 |
| --- | --- | --- |
| Abdominal | Flabby stomach, aerophagia, epigastric discomfort | 5 |
| Nausea |  | 2 |
| Enoral | Hot tongue, strange taste, dry mouth, salivation | 4 |
| Insomnia, fatigue |  | 3 |
| Hiccups |  | 3 |
| Disgust |  | 1 |
| Dizziness |  | 1 |
| Dry mucosa |  | 2 |

W4

| Abdominal | Bubbling in the abdomen, reflux | 2 |
| --- | --- | --- |
| Nausea |  | 2 |
| Enoral | Swallowing pain, gum irritation/bleeding, fogged teeth, furry feeling, brown or white coating on tongue, hot tongue, tightness in throat, sweet taste in mouth | 10 |
| Insomnia |  | 1 |
| Nose | Irritation | 1 |
| Hiccups |  | 1 |
| Whistle on lungs |  | 1 |

W8

| Abdominal | Flatulence, stomach burning, more bowel movements | 4 |
| --- | --- | --- |
| Enoral | Coating on tongue, hot tongue, loss of taste, hoarseness | 4 |
| Reduced sleep quality |  | 1 |
| Sneezing |  | 1 |
| Nervousness |  | 1 |
| Thoracic | Pressure on the chest/heavy chest, increased mucus production | 3 |
| Tremor hand |  | 1 |
| Palpitations |  | 1 |

M6

| Abdominal | Flatulence, burning sensation in the esophagus, soft stools | 4 |
| --- | --- | --- |
| Enoral | Itching tongue, liquid in mouth, sweet taste, different voice, numb tongue, bleeding gums | 9 |
| Nausea |  | 3 |
| Fatigue |  | 1 |
| Nasal obstruction |  | 1 |
| Head pressure |  | 1 |
| Thoracic | Pressure on the chest, pressure on the lungs, scratching in the lungs, wheezing in the lungs, phlegm, dry cough | 9 |

# Discussion

## Dual users

In dual users, symptoms declined less over time than in exclusive e-cigarette users, suggesting that smoking continued to irritate the airways. This conclusion is supported by other longitudinal studies that found higher occurrence of respiratory disease or wheezing in dual users.[5, 6] Showing that dual users and smokers experience more symptoms should be mentioned during smoking cessation councelling as it might be a strong motivator for quitters to stick to e-cigarettes and avoid dual use.

# Appendix Figures

## Appendix Figure 1) Symptoms attributed to smoking at Baseline – Mean (Percentage), N=622


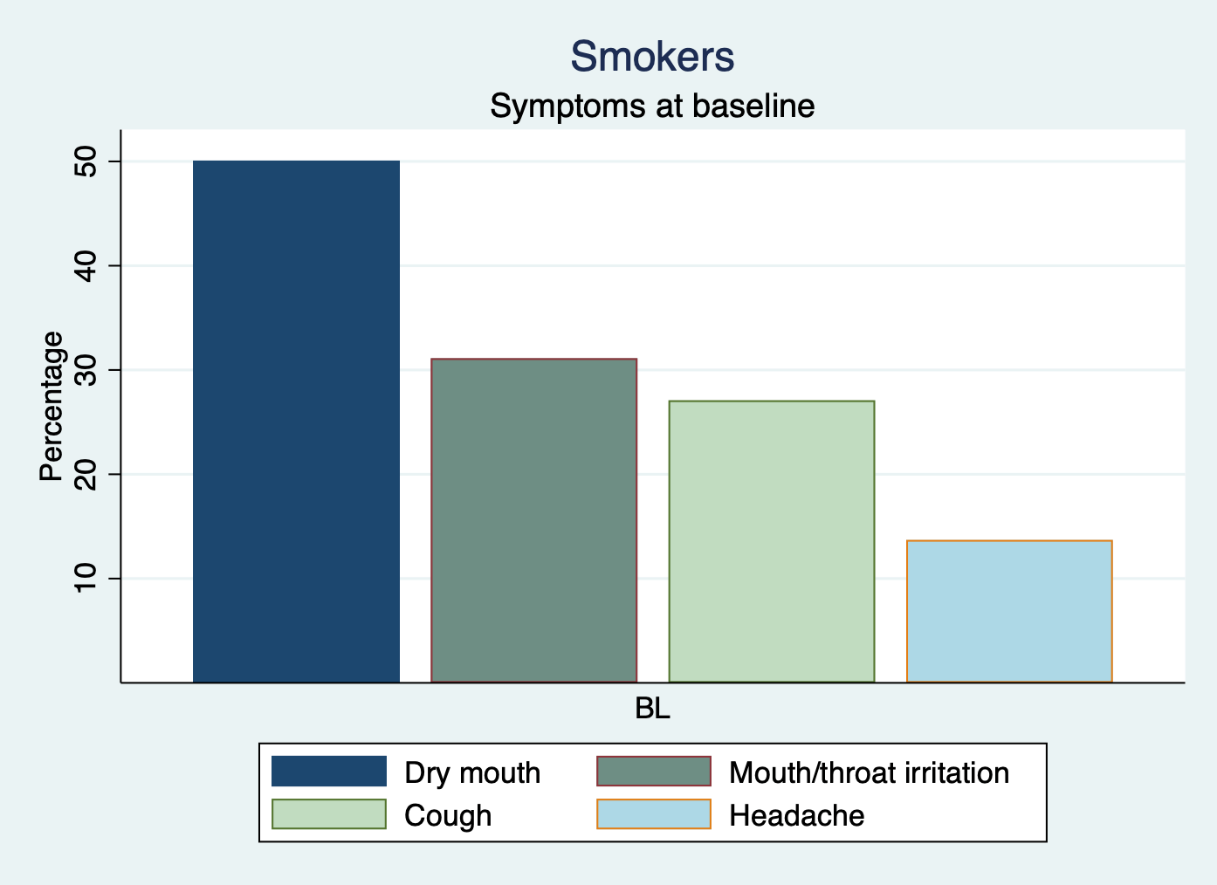


ALT TEXT: Box plots of the distribution of symptoms attributed to smoking at baseline in % among N=622 smokers from the intervention group.

## Appendix Figure 2) Number of participants and their smoking / vaping status over time

Total No. W1: 553; W2: 540; W4: 538; W8: 530; M6: 519


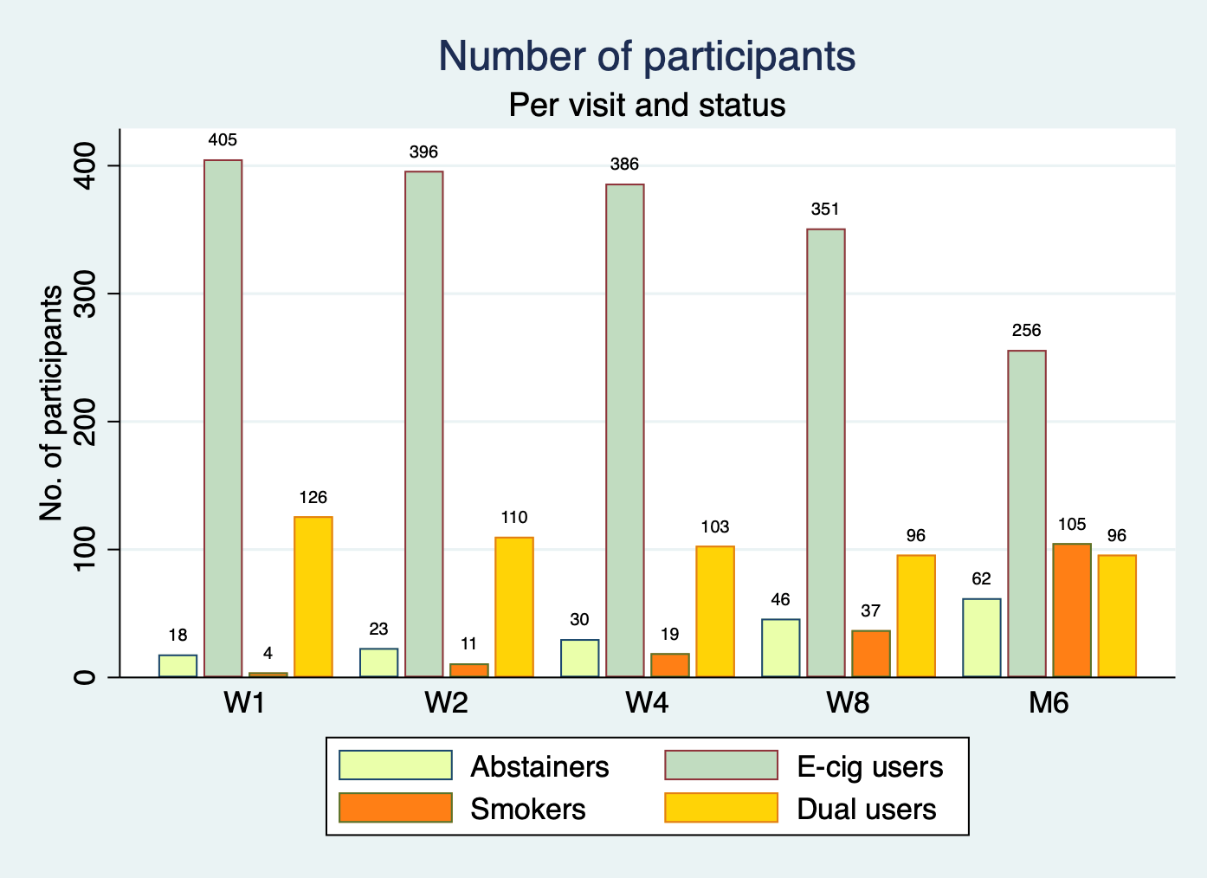


ALT TEXT: Box plots with desription of number of participants at each visit, and their current smoking and vaping status.

## Appendix Figure 3) Symptoms attributed to e-cigarettes in current exclusive e-cigarette users - Percentage

W1 (N=405), W2 (N=396), W4 (N=386), W8 (N=351), M6 (N=256)


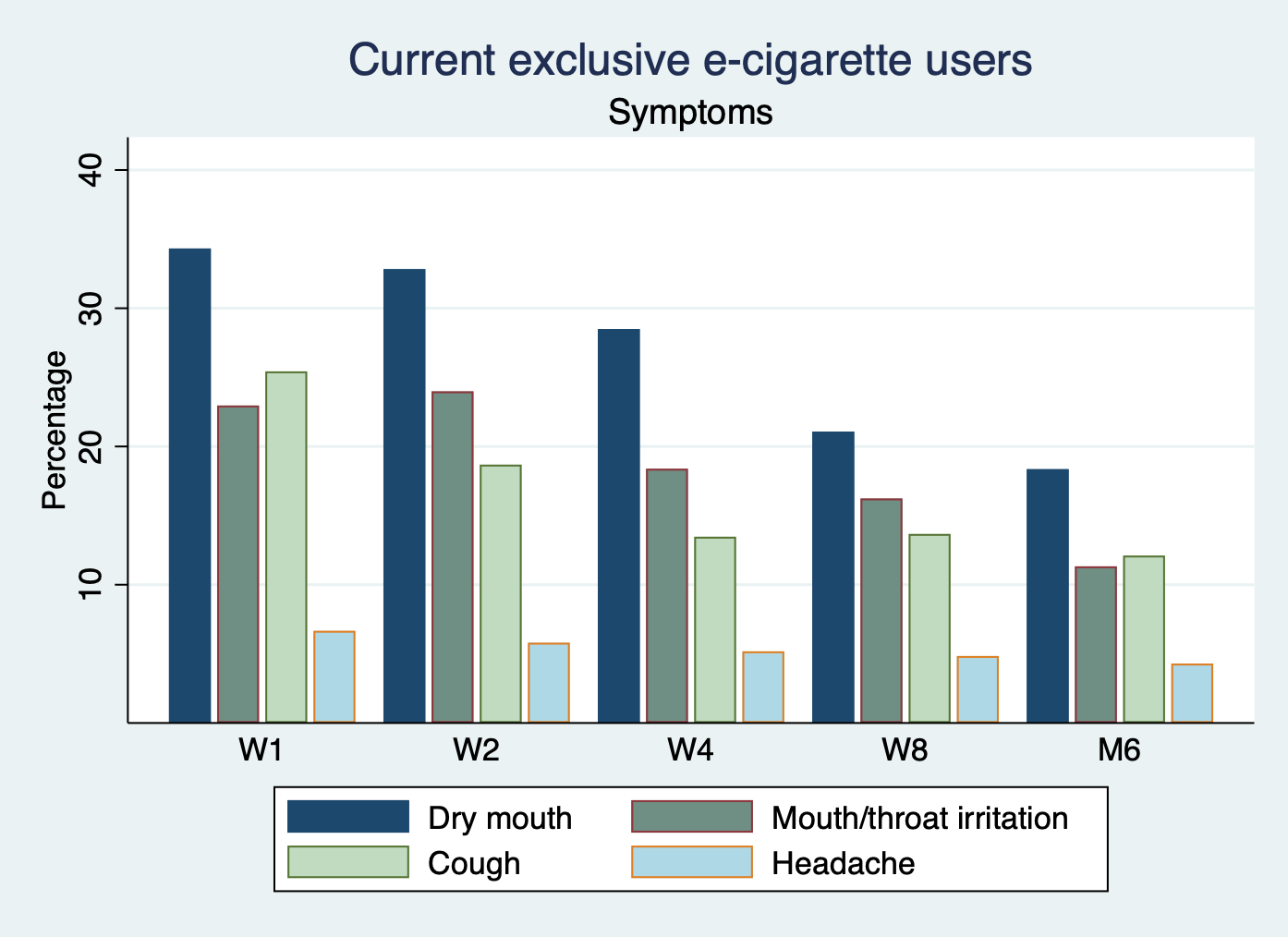


ALT TEXT: Box plots of the distribution of symptoms attributed to e-cigarettes in % of all current exclusive e-cigarette users at every visit.

## Appendix Figure 4) Effect of days of vaping on resolution of dry mouth (results from a marginal structural model)


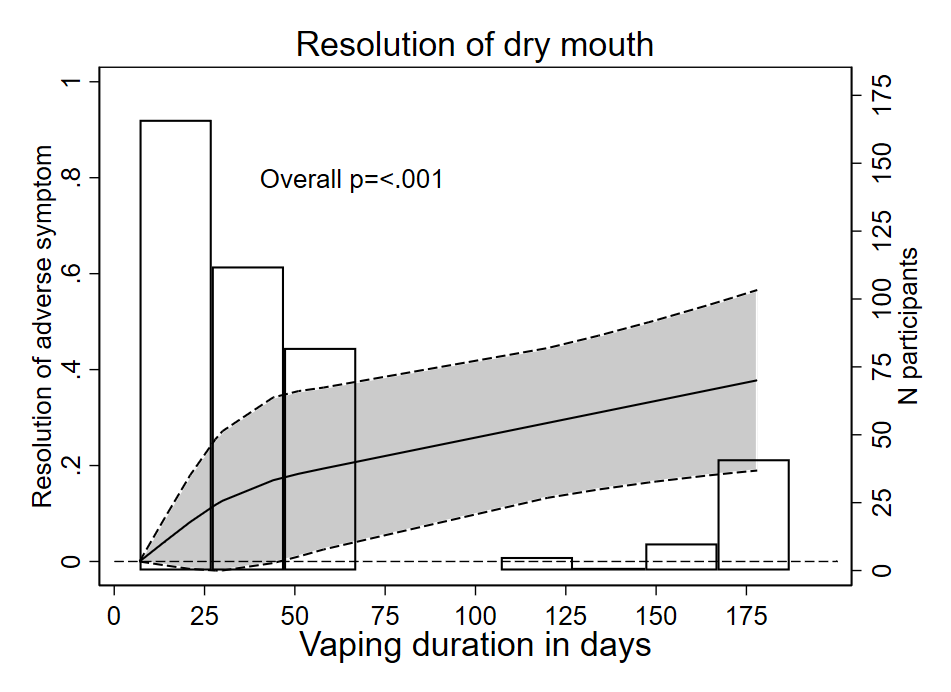


ALT TEXT: Trend and 95% CI of the effect of vaping duration on the resolution of dry mouth in current exclusive e-cigarette users, and box plots showing the number of participants with the corresponding vaping duration in days.

## Appendix Figure 5) Symptoms attributed to e-cigarettes in continuous exclusive e-cigarette users – Percentage

W1 (N=405), W2 (N=349), W4 (N=313), W8 (N=263), M6 (N=181)


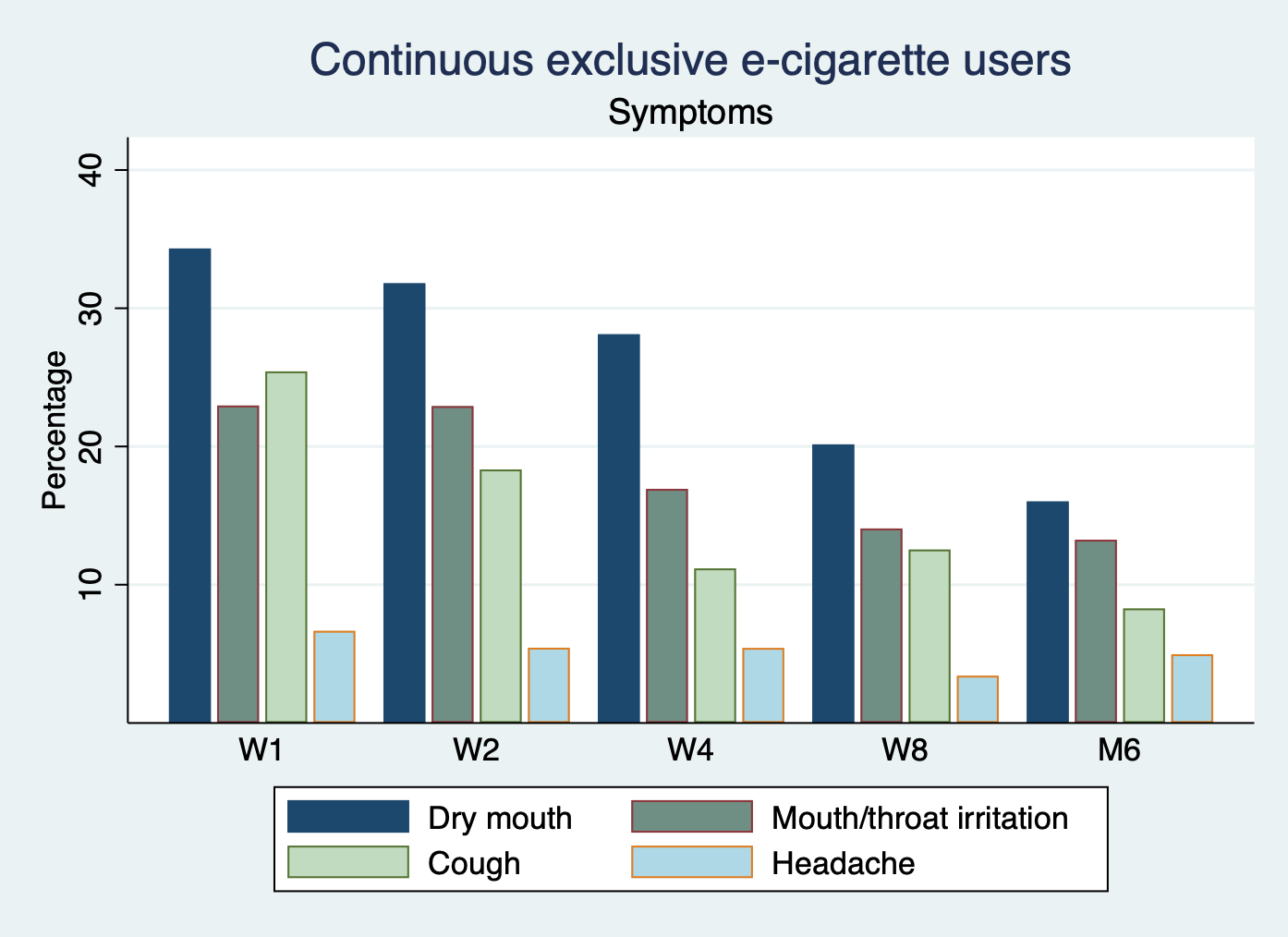


ALT TEXT: Box plots of the distribution of symptoms attributed to e-cigarettes in % of all continuous exclusive e-cigarette users at every visit.

## Appendix Figure 6) Symptoms attributed to cigarettes in exclusive smokers cross-sectionally (control group) – Percentage

BL (N=619), TQD (N=441), W1 (N=198), W2 (N=167), W4 (N=168), W8 (N=187), M6 (N=237)


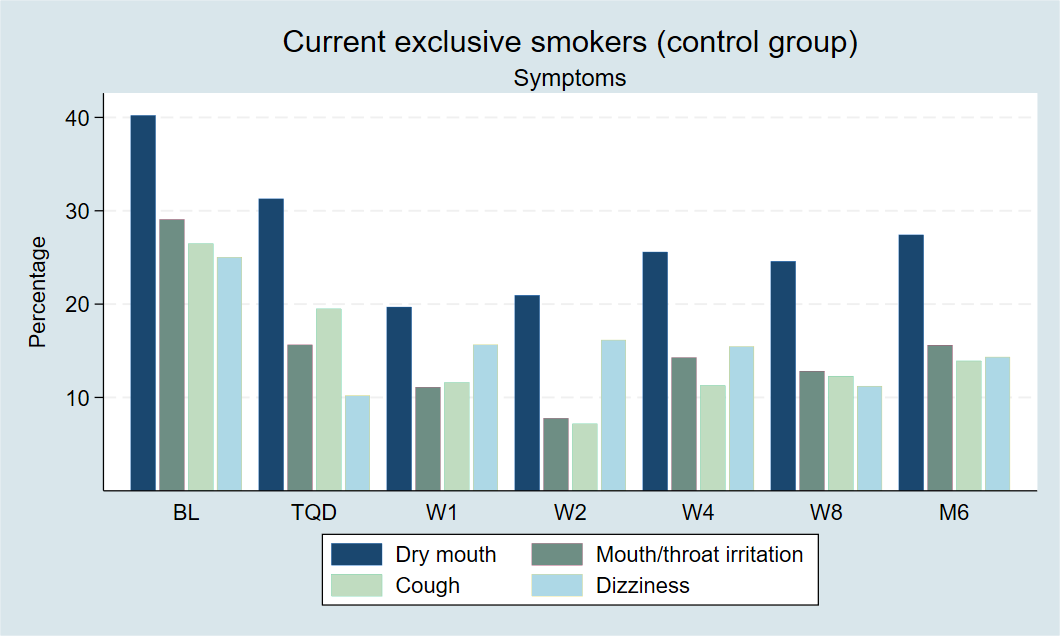


ALT TEXT: Box plots of the distribution of symptoms attributed to cigarette smoking in % of all exclusive smokers at every visit.

## Appendix Figure 7) Symptoms attributed to e-cigarettes in current dual users – Percentage

W1 (N=126), W2 (N=110), W4 (N=103), W8 (N=96), M6 (N=96)


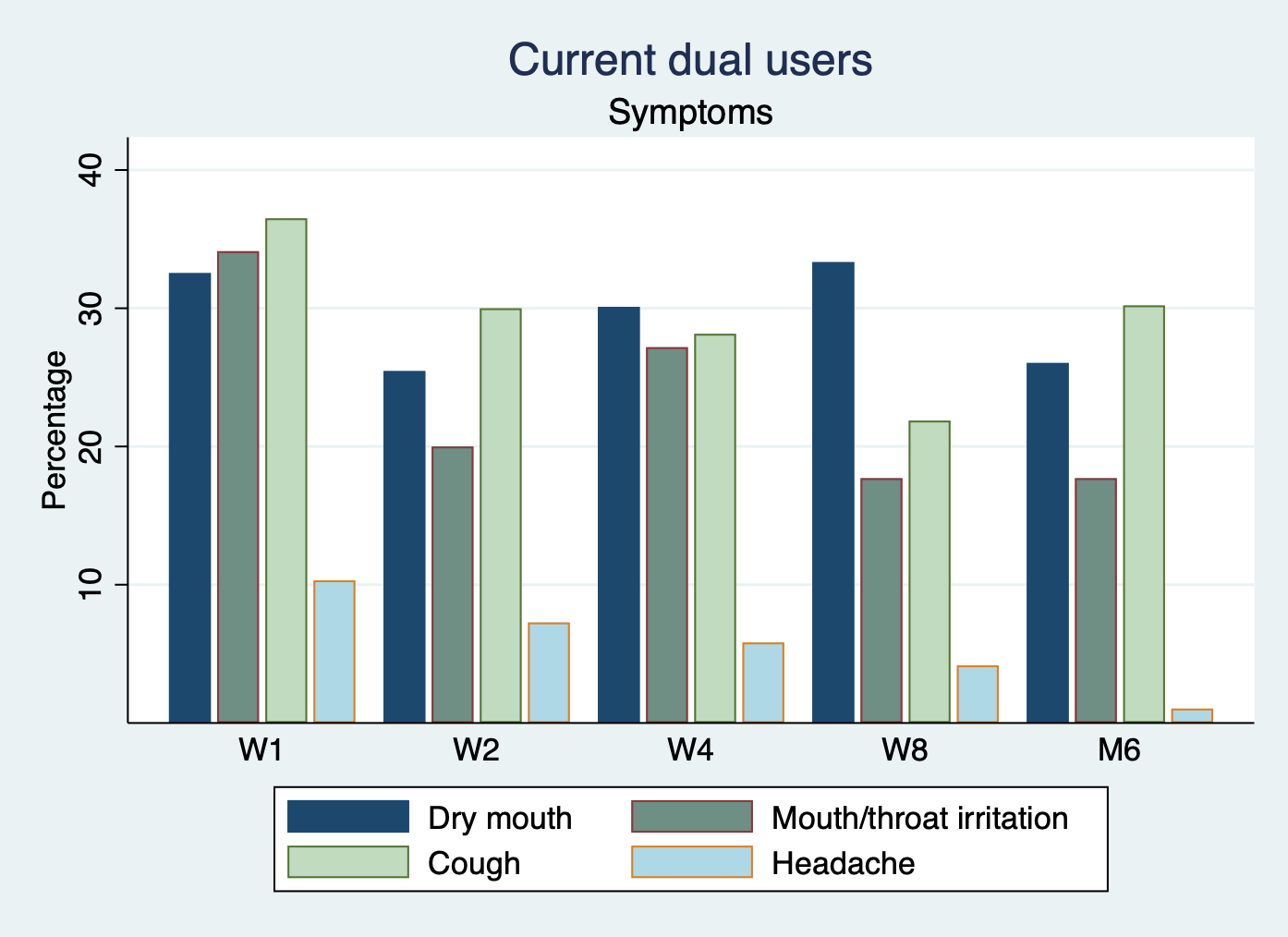


ALT TEXT: Box plots of the distribution of symptoms attributed to e-cigarettes in % of all current dual users at every visit.

## Appendix Figure 8) Symptoms attributed to e-cigarettes in continuous dual users – Percentage

W1 (N=126), W2 (N=69), W4 (N=44), W8 (N=23), M6 (N=11)


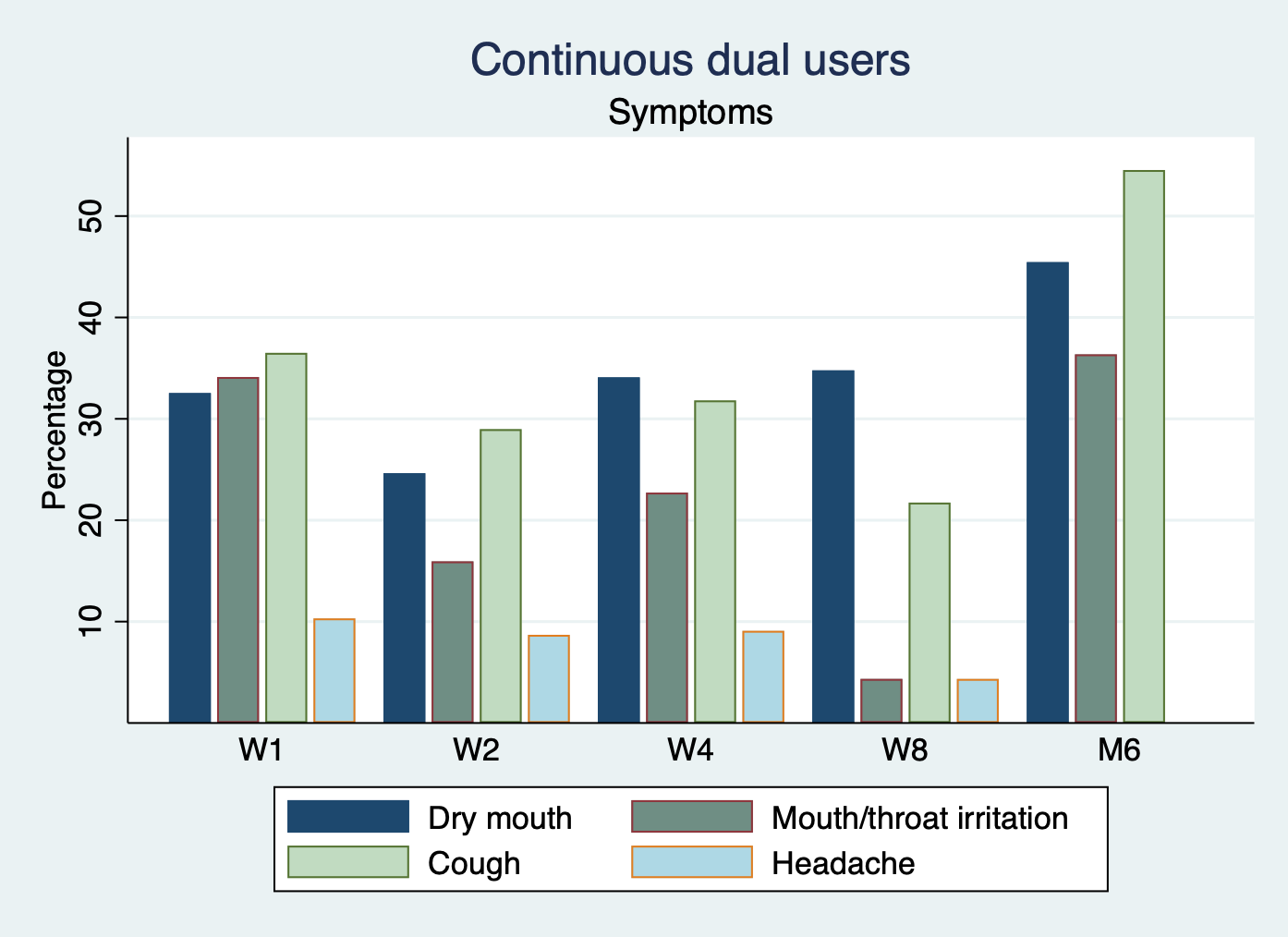


ALT TEXT: Box plots of the distribution of symptoms attributed to e-cigarettes in % of all continuous dual users at every visit.

## Appendix Table 1) Symptoms attributed to e-cigarettes in continuous exclusive e-cigarette users

|  | **Symptoms attributed to cigarettes** | **Symptoms attributed to e-cigarettes** | | | | |
| --- | --- | --- | --- | --- | --- | --- |
|  | **BL**  **N= 622** | **W1** | **W2** | **W4** | **W8** | **M6** |
| **Total continuous exclusive e-cigarette users N** |  | **405** | **349** | **313** | **263** | **181** |
| Dry mouth N (%)  Intensity (p25, p75); [range: 1-6]  Reason to stop (p25, p75); [range: 0-5] | 310 (50%)  3.0 (2.0; 4.0)  1.0 (0.0; 4.0) | 139 (34%)  3.0 (2.0; 4.0) 0.0 (0.0; 1.0) | 111 (32%)  3.0 (2.0; 4.0)  0.0 (0.0; 0.0) | 88 (28%)  3.0 (2.0; 4.0)  0.0 (0.0; 0.5) | 53 (20%)  3.0 (2.0; 4.0)  0.0 (0.0; 0.0) | 29 (16%)  2.0 (1.0; 3.8)  0.0 (0.0; 0.0) |
| Mouth/throat irritation N (%)  Intensity (p25, p75); [range: 1-6]  Reason to stop (p25, p75); [range: 0-5] | 193 (31%)  3.0 (2.0; 4.0)  4.0 (2.0; 5.0) | 93 (23%)  3.0 (2.0; 4.0)  0.0 (0.0; 1.0) | 80 (23%)  3.0 (2.0; 3.2)  0.0 (0.0; 1.0) | 53 (17%)  3.0 (1.5; 4.0)  0.0 (0.0; 2.0) | 37 (14%)  2.0 (1.5; 3.0)  0.0 (0.0; 1.0) | 24 (13%)  3.0 (1.5; 3.0)  0.0 (0.0; 2.0) |
| Cough N (%)  Intensity (p25, p75); [range: 1-6]  Reason to stop (p25, p75); [range: 0-5] | 168 (27%)  3.0 (2.0; 4,0)  5.0 (3.0; 5.0) | 103 (25%)  3.0 (2.0; 3.0)  0.0 (0.0; 1.0) | 64 (18%)  2.0 (1.0; 3.0)  0.0 (0.0; 0.0) | 35 (11%)  2.0 (2.0; 3.0)  0.0 (0.0; 0.0) | 33 (12%)  2.0 (1.5; 3.0)  0.5 (0.0; 2.8) | 15 (8%)  2.0 (1.0; 2.0)  0.0 (0.0; 2.5) |
| Shortness of breath N (%)  Intensity (p25, p75); [range: 1-6]  Reason to stop (p25, p75); [range: 0-5] | 94 (15%)  3.0 (2.0; 5.0)  5.0 (4.0; 5.0) | 13 (3%)  4.0 (2.5; 5.0)  0.0 (0.0; 3.5) | 7 (2%)  2.5 (2.0; 3.0)  0.0 (0.0; 0.0) | 9 (3%)  3.0 (2.5; 4.0)  0.0 (0.0; 3.8) | 4 (2%)  3.0 (2.0; 3.0)  0.0 (0.0; 1.0) | 1 (1%)  n/a  n/a |
| Headache N (%)  Intensity (p25, p75); [range: 1-6]  Reason to stop (p25, p75); [range: 0-5] | 85 (14%)  3.0 (2.0; 4.0)  5.0 (3.0; 5.0) | 27 (7%)  3.0 (2.0; 3.0)  0.0 (0.0; 4.0) | 19 (5%)  4.0 (2.5; 4.0)  1.0 (0.0; 3.0) | 17 (5%)  3.0 (1.5; 4.0)  0.0 (0.0; 1.5) | 9 (3%)  2.0 (1.0; 3.0)  0.0 (0.0; 0.0) | 9 (5%)  2.0 (2.0; 2.0)  0.0 (0.0; 0.0) |
| Dizziness N (%)  Intensity (p25, p75); [range: 1-6]  Reason to stop (p25, p75); [range: 0-5] | 168 (27%)  3.0 (2.0; 4.0)  2.0 (0.0; 5.0) | 16 (4%)  3.0 (3.0; 3.2)  0.0 (0.0; 4.2) | 9 (3%)  3.0 (3.0; 4.2)  0.0 (0.0; 2.5) | 8 (3%)  3.0 (1.0; 4.0)  0.0 (0.0; 2.0) | 4 (2%)  3.0 (3.0; 3.0)  0.0 (0.0; 0.0) | 1 (1%)  n/a  n/a |
| Palpitations/tachycardia N (%)  Intensity (p25, p75); [range: 1-6]  Reason to stop (p25, p75); [range: 0-5] | 61 (10%)  3.5 (2.0; 5.0)  5.0 (2.0; 5.0) | 8 (2%)  3.0 (1.5; 5.5)  0.0 (0.0; 0.5) | 9 (3%)  2.0 (1.8; 3.2)  0.0 (0.0; 2.0) | 10 (3%)  3.0 (3.0; 3.0)  0.0 (0.0; 1.0) | 6 (2%)  2.0 (1.0; 3.0)  0.0 (0.0; 0.0) | 3 (2%)  n/a  n/a |
| Other N (%)  Intensity (p25, p75); [range: 1-6]  Reason to stop (p25, p75); [range: 0-5] | 50 (8%)  4.0 (3.0; 5.0)  4.5 (3.0; 5.0) | 26 (6%)  3.0 (2.8; 4.0)  0.0 (0.0; 2.0) | 17 (5%)  3.0 (2.0; 4.8)  0.0 (0.0; 2.2) | 12 (4%)  4.0 (2.0; 4.5)  0.0 (0.0; 3.0) | 10 (4%)  3.5 (2.2; 4.0)  1.5 (0.0; 3.8) | 10 (6%)  2.0 (2.0; 4.0)  0.0 (0.0; 0.5) |

BL= Baseline, N= number, W=Week, M=Month

## Appendix Table 2) Baseline characteristics of the 622 Participants of the intervention group (subgroup: Week 1 dual users)

| **Characteristics** | **Participants** | **W1 Dual users** |
| --- | --- | --- |
| **Participants** n | 622 | 126 |
| **Age** Median (p25, p75); [range] | 38 (29, 52); [18-79] | 41 (28, 55); [18-73] |
| **Gender** |  |  |
| Women No. (%) | 289 (47) | 62 (49) |
| Men No. (%) | 331 (53) | 64 (51) |
| Other No. (%) | 0 (0) | 0 (0) |
| **Education** |  |  |
| Primary/other/none No. (%) | 50 (8) | 9 (7) |
| Secondary No. (%) | 398 (64) | 89 (71) |
| Tertiary No. (%) | 172 (28) | 28 (22) |
| **Work situation** |  |  |
| Work/house No. (%) | 446 (72) | 83 (66) |
| In formation No. (%) | 57 (9) | 11 (9) |
| Looking for a job/other No. (%) | 117 (19) | 32 (25) |
| **Marital status** |  |  |
| Single No. (%) | 346 (56) | 71 (56) |
| Married/ registered partnership No. (%) | 169 (27) | 29 (23) |
| Widow/ divorced/ dissolved No. (%) | 105 (17) | 26 (21) |
| **Smoking history** |  |  |
| No. of cigarettes smoked daily  Median (p25, p75); [range] | 15 (10, 20); [5 - 60] | 20 (12, 20); [6-60] |
| Age when started smoking  Median (p25, p75); [range] | 16 (15, 18); [8-46] | 17 (15, 19); [11-46] |
| No. of previous quit attempts  Median (p25, p75); [range] | 2 (1, 3); [0 - 50] | 2 (1, 3); [0 - 20] |
| Fagerström score  Median (p25, p75); [range]; Mean (SD) | 4 (3, 6); [0 - 10]; 4.3 (2.3) | 5 (4, 7); [0-10]; 5.2 (2.1) |
| Expired carbon monoxide level  Median (p25, p75); [range] | 20 (13, 29); [0 - 128] | 24 (15, 34); [0 - 128] |
| Packyears*  Median (p25, p75); [range] | 14 (7, 28); [0-132] | 18 (9, 40); [0-132] |
| **Nicotine concentration (mg/ml);**  Mean (SD) |  | 13 |
| **Flavors** |  |  |
| Fruity N (%)* |  | 31 (25%) |
| Tobacco N (%)** |  | 33 (27%) |
| Menthol N (%) |  | 13 (10%) |
| Other flavors N (%)*** |  | 0 (0%) |
| Flavor mix N (%)**** |  | 47 (38%) |
| **Puffs a day** |  |  |
| 1-5 N (%) |  | 2 (1.8%) |
| 6-50 N (%) |  | 56 (50.5%) |
| > 50 N (%) |  | 53 (47.7%) |
| **Substance use** |  |  |
| Alcohol: AUDIT C-Score Median (p25, p75); [range] | 4 (2-5); [0-12] | 4 (1-6); [0-11] |
| Cannabis used in last 30 days No. (%) | 109 (18) | 23 (18) |
| **Health status** |  |  |
| COPD No. (%) | 25 (4) | 10 (8) |
| Chronic bronchitis No. (%) | 22 (4) | 6 (5) |
| Asthma No. (%) | 77 (12) | 17 (13) |
| CAT Score total Median (p25, p75); [range] | 8 (5-12); (0-28) | 9 (5-12); (0-27) |
| CAT Score Question 1 - Cough  Median (p25, p75); [range] | 2 (1-3); (0-5) | 2 (1-3); (0-5) |
| CAT Score Question 2 – Phlegm  Median (p25, p75); [range] | 1 (0-2); (0-5) | 1 (0-2); (0-5) |

## Appendix Table 3) Symptoms attributed to e-cigarettes in dual users cross-sectionally at every visit

|  | **Symptoms attributed to cigarettes** | **Symptoms attributed to e-cigarettes** | | | | |
| --- | --- | --- | --- | --- | --- | --- |
|  | **BL**  **N= 622** | **W1** | **W2** | **W4** | **W8** | **M6** |
| **Total dual users N** |  | **126** | **110** | **103** | **96** | **96** |
| Dry mouth %  Intensity (p25, p75); [range: 1-6]  Reason to stop (p25, p75); [range: 0-5] | 310 (50%)  3.0 (2.0; 4.0)  1.0 (0.0; 4.0) | 41 (33%)  3.5 (2.0; 4.0) 0.0 (0.0; 2.2) | 28 (25%)  3.0 (2.0; 4.0)  0.0 (0.0; 0.5) | 31 (30%)  3.0 (2.0; 5.0)  0.0 (0.0; 1.5) | 32 (33%)  3.0 (2.0; 4.0)  0.0 (0.0; 0.5) | 25 (26%)  3.0 (2.0; 4.0)  0.0 (0.0; 2.0) |
| Mouth/throat irritation %  Intensity (p25, p75); [range: 1-6]  Reason to stop (p25, p75); [range: 0-5] | 193 (31%)  3.0 (2.0; 4.0)  4.0 (2.0; 5.0) | 43 (34%)  4.0 (3.0; 5.0)  0.0 (0.0; 3.0) | 22 (20%)  3.0 (2.0; 4.0)  0.0 (0.0; 3.0) | 28 (27%)  3.0 (2.0; 4.0)  0.0 (0.0; 1.0) | 17 (18%)  3.0 (2.0; 3.0)  0.0 (0.0; 1.0) | 17 (18%)  3.0 (2.0; 3.0)  1.5 (0.0; 2.0) |
| Cough %  Intensity (p25, p75); [range: 1-6]  Reason to stop (p25, p75); [range: 0-5] | 168 (27%)  3.0 (2.0; 4,0)  5.0 (3.0; 5.0) | 46 (37%)  3.0 (2.0; 5.0)  0.0 (0.0; 0.8) | 33 (30%)  2.5 (2.0; 4.8)  0.0 (0.0; 2.5) | 29 (28%)  3.0 (2.0; 4.0)  0.0 (0.0; 3.0) | 21 (22%)  3.0 (3.0; 3.8)  0.0 (0.0; 0.0) | 29 (30%)  2.0 (1.0; 3.0)  0.0 (0.0; 1.5) |
| Shortness of breath N (%)  Intensity (p25, p75); [range: 1-6]  Reason to stop (p25, p75); [range: 0-5] | 94 (15%)  3.0 (2.0; 5.0)  5.0 (4.0; 5.0) | 4 (3%)  4.0 (4.0; 4.0)  3.0 (3.0; 3.0) | 10 (9%)  2.0 (2.0; 4.0)  0.0 (0.0; 1.0) | 4 (4%)  2.0 (2.0; 2.0)  1.0 (1.0; 1.0) | 1 (1%)  n/a  n/a | 3 (3%)  1.0 (1.0; 1.0)  1.0 (1.0; 1.0) |
| Headache %  Intensity (p25, p75); [range: 1-6]  Reason to stop (p25, p75); [range: 0-5] | 85 (14%)  3.0 (2.0; 4.0)  5.0 (3.0; 5.0) | 13 (10%)  2.5 (1.2; 4.5)  0.0 (0.0; 1.5) | 8 (7%)  2.0 (1.2; 2.8)  1.0 (0.0; 4.2) | 6 (6%)  3.0 (3.0; 3.0)  0.0 (0.0; 0.0) | 4 (4%)  3.0 (3.0; 3.0)  1.0 (0.0; 2.0) | 1 (1%)  n/a  n/a |
| Dizziness %  Intensity (p25, p75); [range: 1-6]  Reason to stop (p25, p75); [range: 0-5] | 168 (27%)  3.0 (2.0; 4.0)  2.0 (0.0; 5.0) | 3 (2%)  4.0 (4.0; 4.0)  0.0 (0.0; 0.0) | 8 (7%)  2.0 (2.0; 3.0)  0.0 (0.0; 5.0) | 3 (3%)  n/a  n/a | 2 (2%)  3.0 (3.0; 3.0)  0.0 (0.0; 0.0) | 2 (2%)  n/a  n/a |
| Palpitations/tachycardia %  Intensity (p25, p75); [range: 1-6]  Reason to stop (p25, p75); [range: 0-5] | 61 (10%)  3.5 (2.0; 5.0)  5.0 (2.0; 5.0) | 3 (2%)  2.5 (2.0; 3.0)  0.5 (0.0; 1.0) | 4 (4%)  3.0 (3.0; 3.0)  0.0 (0.0; 0.0) | 5 (5%)  n/a  n/a | 5 (5%)  2.0 (2.0; 2.0)  0.0 (0.0; 0.0) | 1 (1%)  n/a  n/a |
| Other %  Intensity (p25, p75); [range: 1-6]  Reason to stop (p25, p75); [range: 0-5] | 50 (8%)  4.0 (3.0; 5.0)  4.5 (3.0; 5.0) | 4 (3%)  5.0 (3.0; 6.0)  0.0 (0.0; 2.0) | 6 (5%)  4.0 (4.0; 4.0)  0.0 (0.0; 0.0) | 5 (5%)  3.0 (2.0; 4.0)  1.0 (0.0; 2.0) | 3 (3%)  3.0 (3.0; 3.0)  0.0 (0.0; 0.0) | 3 (3%)  2.0 (2.0; 2.0)  0.0 (0.0; 0.0) |

BL= Baseline, N= number, W=Week, M=Month

## Appendix Table 4) Symptoms attributed to e-cigarettes in continuous dual users

|  | **Symptoms attributed to cigarettes** | **Symptoms attributed to e-cigarettes** | | | | |
| --- | --- | --- | --- | --- | --- | --- |
|  | **BL**  **N= 622** | **W1** | **W2** | **W4** | **W8** | **M6** |
| **Total continuous dual users N** |  | **126** | **69** | **44** | **23** | **11** |
| Dry mouth %  Intensity (p25, p75); [range: 1-6]  Reason to stop (p25, p75); [range: 0-5] | 310 (50%)  3.0 (2.0; 4.0)  1.0 (0.0; 4.0) | 41 (33%)  3.5 (2.0; 4.0)  1.0 (0.0; 2.2) | 17 (25%)  3.0 (2.0; 4.0)  0.0 (0.0; 0.0) | 15 (34%)  2.5 (2.0; 5.8)  0.0 (0.0; 0.0) | 8 (35%)  3.0 (3.0; 5.5)  0.0 (0.0; 1.5) | 5 (45%)  3.0 (2.0; 5.0)  0.0 (0.0; 4.0) |
| Mouth/throat irritation %  Intensity (p25, p75); [range: 1-6]  Reason to stop (p25, p75); [range: 0-5] | 193 (31%)  3.0 (2.0; 4.0)  4.0 (2.0; 5.0) | 43 (34%)  4.0 (3.0; 5.0)  0.0 (0.0; 3.0) | 11 (16%)  3.5 (1.8; 4.2)  1.5 (0.0; 3.2) | 10 (23%)  2.5 (1.8; 3.5)  1.5 (0.0; 2.8) | 1 (4%)  2.0 (2.0; 2.0)  0.0 (0.0; 0.0) | 4 (36%)  2.5 (2.0; 3.0)  1.5 (0.0; 3.0) |
| Cough %  Intensity (p25, p75); [range: 1-6]  Reason to stop (p25, p75); [range: 0-5] | 168 (27%)  3.0 (2.0; 4,0)  5.0 (3.0; 5.0) | 46 (37%)  3.0 (2.0; 5.0)  0.0 (0.0; 0.8) | 20 (29%)  2.0 (2.0; 5.0)  0.0 (0.0; 3.0) | 14 (32%)  3.5 (1.8; 6.0)  0.0 (0.0; 5.0) | 5 (22%)  4.0 (4.0; 4.0)  0.0 (0.0; 0.0) | 6 (55%)  3.0 (1.0; 5.0)  1.5 (0.0; 3.0) |
| Shortness of breath N (%)  Intensity (p25, p75); [range: 1-6]  Reason to stop (p25, p75); [range: 0-5] | 94 (15%)  3.0 (2.0; 5.0)  5.0 (4.0; 5.0) | 4 (3%)  4.0 (4.0; 4.0)  3.0 (3.0; 3.0) | 8 (12%)  2.0 (1.5; 4.0)  0.0 (0.0; 0.5) | 2 (5%)  2.0 (2.0; 2.0)  1.0 (1.0; 1.0) | 0 (0%)  n/a  n/a | 0 (0%)  n/a  n/a |
| Headache %  Intensity (p25, p75); [range: 1-6]  Reason to stop (p25, p75); [range: 0-5] | 85 (14%)  3.0 (2.0; 4.0)  5.0 (3.0; 5.0) | 13 (10%)  2.5 (1.2; 4.5)  0.0 (0.0; 1.5) | 6 (9%)  1.5 (1.0; 2.0)  0.0 (0.0; 0.0) | 4 (9%)  3.0 (3.0; 3.0)  0.0 (0.0; 0.0) | 1 (4%)  3.0 (3.0; 3.0)  2.0 (2.0; 2.0) | 0 (0%)  n/a  n/a |
| Dizziness %  Intensity (p25, p75); [range: 1-6]  Reason to stop (p25, p75); [range: 0-5] | 168 (27%)  3.0 (2.0; 4.0)  2.0 (0.0; 5.0) | 3 (2%)  4.0 (4.0; 4.0)  0.0 (0.0; 0.0) | 5 (7%)  3.0 (3.0; 3.0)  0.0 (0.0; 0.0) | 2 (5%)  n/a  n/a | 0 (0%)  n/a  n/a | 0 (0%)  n/a  n/a |
| Palpitations/tachycardia %  Intensity (p25, p75); [range: 1-6]  Reason to stop (p25, p75); [range: 0-5] | 61 (10%)  3.5 (2.0; 5.0)  5.0 (2.0; 5.0) | 3 (2%)  2.5 (2.0; 3.0)  0.5 (0.0; 1.0) | 3 (4%)  3.0 (3.0; 3.0)  0.0 (0.0; 0.0) | 2 (5%)  n/a  n/a | 1 (4%)  n/a  n/a | 0 (0%)  n/a  n/a |
| Other %  Intensity (p25, p75); [range: 1-6]  Reason to stop (p25, p75); [range: 0-5] | 50 (8%)  4.0 (3.0; 5.0)  4.5 (3.0; 5.0) | 4 (3%)  5.0 (3.0; 6.0)  0.0 (0.0; 2.0) | 5 (7%)  4.0 (4.0; 4.0)  0.0 (0.0; 0.0) | 3 (7%)  4.0 (4.0; 4.0)  2.0 (2.0; 2.0) | 0 (0%)  n/a  n/a | 0 (0%)  n/a  n/a |

BL= Baseline, N= number, W=Week, M=Month;

## Appendix Table 5) Symptoms attributed to cigarettes in exclusive smokers cross-sectionally (control group)

|  | **BL**  **N = 619** | **W1**  **N = 198** | **W2**  **N = 167** | **W4**  **N = 168** | **W8**  **N = 187** | **M6**  **N = 237** |
| --- | --- | --- | --- | --- | --- | --- |
| Dry mouth N (%) | 249 (40%) | 39 (20%) | 35 (21%) | 43 (26%) | 46 (25%) | 65 (27%) |
| Mouth/throat irritation N (%) | 180 (29%) | 22 (11%) | 13 (8%) | 24 (14%) | 24 (13%) | 37 (16%) |
| Cough % N (%) | 164 (26%) | 23 (12%) | 12 (7%) | 19 (11%) | 23 (12%) | 33 (14%) |
| Dizziness N (%) | 155 (25%) | 31 (16%) | 27 (16%) | 26 (15%) | 21 (11%) | 34 (14%) |

## Appendix Table 6) Choice of e-liquids (flavors/ nicotine concentrations) and puffs/day in exclusive e-cigarette users

|  | **TQD**  **N = 124** | **W1**  **N = 405** | **W2**  **N = 396** | **W4**  **N = 386** | **W8**  **N = 351** | **M6**  **N = 256** |
| --- | --- | --- | --- | --- | --- | --- |
| **Nicotine concentration (mg/ml)**  Mean (SD) | 14 (5) | 13 (5) | 12 (5) | 11 (5) | 10 (5) | 6 (4) |
| **Flavors** |  |  |  |  |  |  |
| Fruity * No (%) | 32 (26%) | 101 (25%) | 116 (29%) | 115 (30%) | 105 (30%) | 75 (31%) |
| Tobacco** No (%) | 34 (28%) | 97 (24%) | 83 (21%) | 84 (22%) | 77 (22%) | 53 (22%) |
| Menthol No (%) | 11 (9%) | 35 (9%) | 46 (12%) | 43 (11%) | 42 (12%) | 40 (16%) |
| Other flavors *** No (%) | 2 (2%) | 1 (<1%) | 2 (<1%) | 5 (1%) | 5 (1%) | 10 (4%) |
| Flavor mix **** No (%) | 43 (35%) | 169 (42%) | 147 (37%) | 137 (36%) | 119 (34%) | 66 (27%) |
| **Puffs a day** |  |  |  |  |  |  |
| 1-5 No. (%) | 3 (3%) | 7 (2%) | 5 (1%) | 7 (2%) | 6 (2%) | 8 (3%) |
| 6-50 No. (%) | 64 (55%) | 143 (37%) | 134 (35%) | 124 (34%) | 114 (34%) | 81 (33%) |
| > 50 No. (%) | 50 (43%) | 238 (61%) | 241 (63%) | 236 (64%) | 215 (64%) | 157 (64%) |

* Fruity flavors: green apple, raspberry, red fruit, or other fruity flavors (cherry, blood orange, berry, lemon, apricot, peach, nectarine, blueberry, jackfruit, passion fruit, grenadine, mango, and grapes).

** Tobacco flavors: tobacco FR-M, tobacco FR4 or another tobacco flavor.

*** Other flavors: chai, sparkling voddo juic, marchmello, eisenbeer, piña colada, bloody summer, hazelnut, drunken pudding, cucumber, coffee, caramel, and emmentaler drachenfrucht.

## Appendix Table 7) Main Symptoms by flavoring use

|  | **Symptoms attributed to cigarettes** | **Symptoms attributed to e-cigarettes** | | | | | |
| --- | --- | --- | --- | --- | --- | --- | --- |
|  | **BL**  **N= 622** | **W1** | **W2** | **W4** | **W8** | **M6** | **p-value*** |
| **Total exclusive e-cigarette users N**  Fruity flavor  Tobacco flavor  Menthol flavor  Other flavor  Flavor mix  Missing flavor |  | **405**  101  97  35  1  169  2 | **396**  116  83  46  2  147  2 | **386**  115  84  43  5  137  2 | **351**  105  77  42  5  119  3 | **256**  75  53  40  10  66  12 | **n/a** |
| **Dry mouth**, overall  Fruity flavor  Tobacco flavor  Menthol flavor  Other flavor  Flavor mix  Missing flavor | 310 (50%)  -  -  -  -  -  - | 139 (34%)  39 (39%)  33 (34%)  9 (26%)  0 (0%)  58 (34%)  0 (0%) | 130 (33%)  34 (29%)  29 (35%)  14 (30%)  1 (50%)  52 (35%)  0 (0%) | 110 (28%)  29 (25%)  23 (27%)  9 (21%)  2 (40%)  47 (34%)  0 (0%) | 74 (21%)  23 (22%)  13 (17%)  12 (29%)  1 (20%)  25 (21%)  0 (0%) | 47 (18%)  17 (23%)  9 (17%)  10 (25%)  0 (0%)  10 (15%)  1 (8%) | <0.001  0.056  0.019  0.878  0.231  0.003  - |
| **Mouth/throat irritation**, overall  Fruity flavor  Tobacco flavor  Menthol flavor  Other flavor  Flavor mix  Missing flavor | 193 (31%)  -  -  -  -  -  - | 93 (23%)  24 (24%)  19 (20%)  6 (17%)  1 (100%)  43 (25%)  0 (0%) | 95 (24%)  26 (22%)  25 (30%)  8 (17%)  1 (50%)  34 (23%)  1 (50%) | 71 (18%)  17 (15%)  18 (21%)  10 (23%)  1 (20%)  25 (18%)  0 (0%) | 57 (16%)  16 (15%)  12 (16%)  3 (7%)  0 (0%)  25 (21%)  1 (33%) | 29 (11%)  8 (11%)  5 (9%)  3 (8%)  1 (10%)  9 (14%)  3 (25%) | <0.001  0.094  0.040  0.161  0.104  0.277  - |
| **Cough**, overall  Fruity flavor  Tobacco flavor  Menthol flavor  Other flavor  Flavor mix  Missing flavor | 168 (27%)  -  -  -  -  -  - | 103 (25%)  31 (31%)  17 (18%)  7 (20%)  1 (100%)  47 (28%)  0 (0%) | 74 (19%)  21 (18%)  15 (18%)  10 (22%)  1 (50%)  26 (18%)  1 (1%) | 52 (13%)  15 (13%)  15 (18%)  6 (14%)  1 (20%)  15 (11%)  0 (0%) | 48 (14%)  16 (15%)  9 (12%)  4 (10%)  0 (0%)  19 (16%)  0 (0%) | 31 (12%)  11 (15%)  4 (8%)  1 (2%)  2 (20%)  11 (17%)  2 (17%) | <0.001  0.008  0.338  0.072  0.203  0.004  - |

*we provide N (%), p-values are from an omnibus test over visits.

## Appendix Table 8) Comparison between participants lost to follow up versus not lost to follow up

|  | **Not lost to follow up** | **Lost to follow up** | **Total** | **p-Wert** |
| --- | --- | --- | --- | --- |
|  | N=550 (89%) | N=70 (11%) | N=622  (100.0%) |  |
| **Gender** |  |  |  |  |
| Men No. (%) | 291 (53%) | 40 (57%) | 331 (53%) | 0.504 |
| Women No. (%) | 259 (47%) | 30 (43%) | 289 (47%) |  |
| **Age mean** | 40 (14) | 37(13) | 40 (14) | 0.090 |
| **Education** |  |  |  |  |
| Primary/other/none No. (%) | 39 (7%) | 11 (16%) | 50 (8%) | 0.011* |
| Secondary No. (%) | 351 (64%) | 47 (67%) | 398 (64%) |  |
| Tertiary No. (%) | 160 (29%) | 12 (17%) | 172 (28%) |  |
| **Work situation** |  |  |  |  |
| Work/house No. (%) | 403 (73%) | 43 (61%) | 446 (72%) | 0.115 |
| In formation No. (%) | 48 (9%) | 9 (13%) | 57 (9%) |  |
| Looking for a job/other No. (%) | 99 (18%) | 18 (26%) | 117 (19%) |  |
| **No. of cigarettes smoked daily**  Median (p25, p75); [range] | 15 (10, 20); [5-45] | 19 (10, 20); [5-45] | 15 (10, 20); [5-45] | 0.095 |
| **Cannabis used in last 30 days** |  |  |  |  |
| No No. (%) | 463 (84%) | 48 (69%) | 511 (82%) | 0.001* |
| Yes No. (%) | 87 (16%) | 22 (31%) | 109 (18%) |  |
| **Alcohol: AUDIT C-Score** Median (p25, p75); [range] | 4 (2-5); [0-12] | 4 (2-6); [0-12] | 4 (2-5); [0-12] | 0.471 |
| **Current disease** |  |  |  |  |
| No No. (%) | 527 (96%) | 69 (99%) | 596 (96%) | 0.261 |
| Yes No. (%) | 23 (4%) | 1 (1%) | 24 (4%) |  |
| **COPD** |  |  |  |  |
| No No. (%) | 529 (96%) | 66 (94%) | 595 (96.0%) | 0.448 |
| Yes No. (%) | 21 (4%) | 4 (6%) | 25 (4.0%) |  |
| **Asthma** |  |  |  |  |
| No No. (%) | 482 (88%) | 61 (87%) | 543 (87%) | 0.906 |
| Yes No. (%) | 69 (12%) | 9 (13%) | 77 (12%) |  |

**People with lower educational status and people not using cannabis were more often lost to follow up.*

##

# References

1. Auer, R., et al., *Electronic Nicotine-Delivery Systems for Smoking Cessation.* N Engl J Med, 2024. **390**(7): p. 601-610.

2. Mosimann, A.F., et al., *E-liquid flavors and nicotine concentration choices over 6 months after a smoking cessation attempt with ENDS: Secondary analyses of a randomized controlled trial.* Tob Prev Cessat, 2025. **11**.

3. Robins, J.M., M.A. Hernan, and B. Brumback, *Marginal structural models and causal inference in epidemiology.* Epidemiology, 2000. **11**(5): p. 550-60.

4. Fewell, Z., et al., *Controlling for time-dependent confounding using marginal structural models.* Stata Journal, 2004. **4**(4): p. 402-420.

5. Bhatta, D.N. and S.A. Glantz, *Association of E-Cigarette Use With Respiratory Disease Among Adults: A Longitudinal Analysis.* Am J Prev Med, 2020. **58**(2): p. 182-190.

6. Sanchez-Romero, L.M., et al., *Assessment of Electronic Nicotine Delivery Systems With Cigarette Use and Self-reported Wheezing in the US Adult Population.* JAMA Netw Open, 2023. **6**(4): p. e236247.
